# Development of CAR T Cell Therapy in Children—A Comprehensive Overview

**DOI:** 10.3390/jcm11082158

**Published:** 2022-04-12

**Authors:** Michael Boettcher, Alexander Joechner, Ziduo Li, Sile Fiona Yang, Patrick Schlegel

**Affiliations:** 1 Department of Pediatric Surgery, University Medical Centre Mannheim, University of Heidelberg, 69117 Heidelberg, Germany; michael.boettcher@umm.de; 2 School of Medical Sciences, Faculty of Medicine and Health, University of Sydney, Sydney 2006, Australia; ajoechner@cmri.org.au; 3 Cellular Cancer Therapeutics Unit, Children’s Medical Research Institute, Sydney 2145, Australia; cli@cmri.org.au (Z.L.); syang@cmri.org.au (S.F.Y.); 4 Department of Pediatric Hematology and Oncology, Westmead Children’s Hospital, Sydney 2145, Australia

**Keywords:** evolution of CAR T cells, FDA-approved CAR products, TcR versus CAR, limitations and complications of CAR T cell therapy, future directions of CAR T cell therapy

## Abstract

CAR T cell therapy has revolutionized immunotherapy in the last decade with the successful establishment of chimeric antigen receptor (CAR)-expressing cellular therapies as an alternative treatment in relapsed and refractory CD19-positive leukemias and lymphomas. There are fundamental reasons why CAR T cell therapy has been approved by the Food and Drug administration and the European Medicines Agency for pediatric and young adult patients first. Commonly, novel therapies are developed for adult patients and then adapted for pediatric use, due to regulatory and commercial reasons. Both strategic and biological factors have supported the success of CAR T cell therapy in children. Since there is an urgent need for more potent and specific therapies in childhood malignancies, efforts should also include the development of CAR therapeutics and expand applicability by introducing new technologies. Basic aspects, the evolution and the drawbacks of childhood CAR T cell therapy are discussed as along with the latest clinically relevant information.

## 1. Introduction

CAR T cell therapy has revolutionized immunotherapy in the last decade with the successful establishment of chimeric antigen receptor (CAR)-expressing cellular therapies as an alternative treatment in relapsed and refractory (r/r) homogeneously CD19-positive leukemias and lymphomas [1,2,3]. There are fundamental reasons why CAR T cell therapy has been approved by the Food and Drug administration (FDA) in the USA and the European Medicines Agency (EMA) for pediatric and young adult patients, as well as adult patients whose clinical data usually pave the way for translation of novel therapies into the clinic for children. Commonly, novel therapies are developed for the larger adult patient cohort, and then adapted for pediatric use, due to regulatory and commercial reasons [4,5]. Both strategic and biological factors have supported the development of CAR T cell therapy in children. The higher clinical relevance of CD19-positive malignancies in children compared to adults is one of the pivotal factors. B-cell acute lymphoblastic leukemia (B-ALL) is the most common pediatric malignancy, with a prevalence of up to 25% of cancers in all childhood cancers [6]. In contrast, the prevalence of all cancers in adults is below 0.5%, and B-cell non-Hodgkin’s lymphoma (NHL) represents approximately 3.6% of adult cancers [7,8]. Despite the unprecedented success story of ALL treatment in childhood, with 5 year overall survival rates exceeding 90% in contemporary treatment optimization studies [9], prognosis for r/r patients and patients with high-risk predispositions is still dismal [10]. Therefore, there is an urgent need for improved and more specific therapies in r/r ALL to reduce the adverse event profile and prolong survival. Furthermore, the susceptibility of B-ALL to CAR T cell therapy is significantly higher [2] than that of chronic lymphoblastic leukemia (CLL) [11] and a broad variety of B-lineage-derived lymphomas [12]. 

In general, pediatric ALL is an unmatched success story in cancer treatment, with high overall survival (OS) rates throughout the Western world, drastically increasing from no chance of survival in the 1950s, ~10% OS in the 1960s, ~40% OS in the 1970s, ~65% in the 1980s, to survival rates above 90% today [9]. The main reason for the excellent survival rates is the sophisticated chemotherapy protocols that have been initiated and optimized over the last seven decades [13]. Moreover, major advances have been achieved with the development and improvement of allogeneic hematopoietic stem cell transplantation (allo-HSCT) [14] and immunotherapy with the bispecific T cell engager therapy (BiTE) blinatumomab (CD3XCD19) [15,16], which is currently trialed in patients with precursor B-ALL as an alternative to conventional intensive and toxic chemotherapies, and in patients who are at high risk of relapse post chemotherapy in the clinical trial AIEOP-BFM ALL 2017 (NCT03643276). 

CD19-CAR T cell therapy has been a medical breakthrough in the treatment of pediatric ALL, demonstrated by its outstanding clinical success, which exceeds previous therapies including allo-HSCT and blinatumomab treatment in r/r patients considered to be incurable with a shortened life expectancy [2,17]. CD19-targeted CAR-expressing T cells (CD19-CAR-T) were able to cure pediatric patients with a single-agent infusion trialed as the last resort after blinatumomab therapy [2]. Subsequent exploration of CD19-CAR-T cell treatment also demonstrated success in r/r ALL patients post allo-HSCT after infusion of true-allogeneic CD19-CAR T cells (donor-derived) [18] and pseudo-allogeneic (posttransplant recipient-derived) CD19-CAR T cells [19]. In the landmark clinical trials NCT01626495 and NCT01029366, autologous CD19-CAR-T treatment resulted in a high response rate (90% complete remission induction) and a 50% long-term event-free survival, despite recruitment of a limited number (N = 25) of patients [2]. These unprecedented clinical data in CAR T cell trials have led to the FDA approval of the first CD19-CAR-T cell therapy in children and young adults with B-ALL in 2017.

To date, the clinical development of CAR T cell therapy has only been successful (beyond case reports) in B-lineage-derived acute and chronic hematologic malignancies [2,3,20]. The overwhelming and convincing clinical benefits over other existing treatments in r/r B-lineage malignancies have led to FDA and/or EMA approvals of more CD19-, as well as BCMA-targeted CAR therapeutics (Table 1). To date, r/r B-ALL [21], r/r diffuse large B-cell lymphoma (DLBCL) [22,23], r/r follicular lymphoma (FL) [22,23], mantle cell lymphoma (MCL) [24] and r/r multiple myeloma (MM) [25] can be treated successfully with the FDA-approved CAR products. Amongst the four approved CD19-CAR-T cell products, data to support the choice of the optimal therapy for different B-lineage-derived cancers are lacking, and further evaluation in clinical trials will be required to identify a treatment algorithm that enables timely and optimal use of these CAR T cell treatments [26]. The clinical success of CD19-CAR-T cell therapy has led to great expectations of translating CAR T cell strategies beyond B-lineage malignancies.

The future directions of CAR T cell therapy are to develop advanced CAR technologies to overcome the current limitations in CAR-mediated immunotherapy, which are toxicities and limited or lack of efficacy. Toxicities that arise from CAR T cell therapy include acute life-threatening complications, such as cytokine release syndrome (CRS) [27,28], immune effector cell-associated neurotoxicity syndrome (ICANS) [28] and mid-term and long-term side effects caused by profound B-cell aplasia that requires human IgG substitution to prevent severe infectious complications [29]. 

The long-term efficacy of CAR T cell therapy may be improved by addressing treatment failure due to antigen escape in pediatric patients. Relapses in approximately 25% of patients can be accounted for by antigen loss or downregulation, lineage switch or primary target antigen heterogeneity [30], lack of persistence and fitness of cells and resistance to CAR T cell therapy due to immunosuppressive factors such as immune checkpoint inhibition (PD-1), poor trafficking and tumor infiltration [31]. Chen et al. were able to identify gene signatures of TCF7 and IFN response genes in CD19-CAR-T cell products for pediatric patients to predict CAR T cell persistence, which is associated with long-term survival. Constant IFN signaling negatively impacts on CAR T cell performance. Thus, elucidating the underlying molecular determinants of clinical CAR T cell function may facilitate improving the clinical efficacy of CAR T cell therapy by adapting CAR T cell manufacturing to induce a favorable gene expression profile or by introducing novel genetic modifications [32]. Moreover, T cell exhaustion and senescence impact on the performance of T cells and CAR T cells. T cell senescence and restoration of T cell function are determinants of longevity and anticancer function but seem to be more evident in elderly patients than in children [33]. In solid cancers, immunosuppressive ligands and soluble factors, low oxygen and glucose levels in the tumor microenvironment (TME) have been identified to be the most important factors that limit the anticancer activity of CAR T therapeutics [34]. 

This review will provide insights into the molecular architecture and function of CAR T cells and touch on new advanced CAR technologies, as well as elucidating the importance of target antigens, the historic development of CAR technology and T cell receptor immunology. Further, the FDA/EMA-approved products will be reviewed to introduce the state-of-the-art CAR T cell therapy in children hitherto, covering major complications, relapse patterns and challenges of current CAR T cell concepts. 

## 2. Methods

We used open-source medical and clinical trial databases including PubMed and Clinicaltrials.gov (accessed on 25 March 2022) to extract the information presented and discussed in this review article.

## 3. Molecular Architecture of CAR Receptors

CAR T cells are artificially generated transgenic cells that express a hybrid in silico designed de novo dimeric immune receptor. The basic architecture of CAR receptors is an extracellular antigen recognition domain, a spacer domain, a transmembrane domain, and an intracellular signaling domain [35]. Each domain of a CAR receptor has been intensively studied and variations have been designed and established successfully. It is noteworthy that critical steps in the development of CAR receptors were necessary to make CAR T cells potent therapeutics being capable of curing patients [36,37]. 

**Figure 1:**
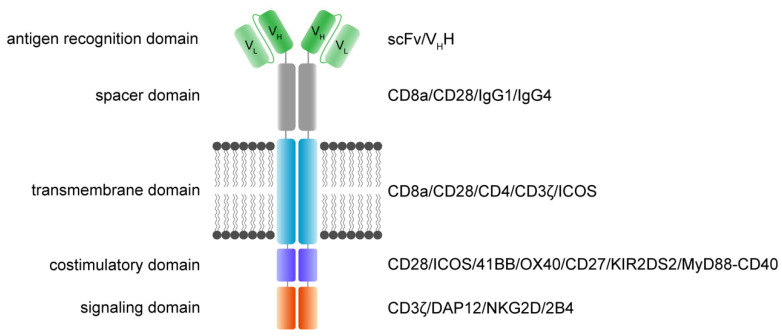
Functional modules of CAR receptors.

The main function and idea of CAR receptors are obviously to enable immune effector cells such as T cells and NK cells to be specifically redirected to cancer cells overexpressing the target antigen in a major histocompatibility complex (MHC)-independent manner [38,39]. scFv-based CAR receptors may also be constructed to target peptides presented by the MHC, for instance HLA-A2/NY-ESO-1 [40]. In Figure 1, the CAR architecture is illustrated and indicates established domain-variations.

A schematic illustration of a second-generation CAR receptor. CAR receptors are comprised of several modules indicated in different colors—the antigen recognition domain, which usually consists of an antibody-derived scFv or V_H_H, the spacer domain of variable length, configuration, and flexibility, connecting the antigen recognition domain to the transmembrane domain. The transmembrane domain robustly anchors the CAR in the phospholipid bilayer cell membrane and is linked to the intracellular parts of the artificial immune receptor. Thus, another important role of the transmembrane domain is to facilitate the mechanic signal transduction into the cell. The intracellular costimulatory domains and signaling domain transform the activation signal via a signaling cascade into the cell to activate downstream signaling that results in various effector functions such as cytolysis, cytokine secretion and proliferation. scFv: single-chain variable fragment; V_H_H: heavy chain variable fragment of a single-domain antibody; V_L_: variable fragment of the light chain; V_H_: variable fragment of the heavy chain. 

A CAR is a modular structure typically consisting of an extracellular antigen-binding domain linked by a spacer region to a transmembrane domain, attached to one or more intracellular activation domains. In general, every subunit of a CAR can significantly change the properties and function of the CAR receptor. CAR design has evolved over the last three decades, with the goal to improve CAR T cell efficacy, persistence, and safety. 

The extracellular recognition domain in most CAR receptors is derived from the variable segments of the antibody light and heavy chains. They are constructed in line with peptide linkers [35,38] to assemble in a single-chain variable fragment (scFv) format. In general, scFvs are less stable in their configuration compared to the Fab region of antibodies [41]. Most antibodies in the past were generated by immunization of mice [42]. Today, fully human antibodies can be generated [43]. Single-domain VH binders (sdFv) based on human libraries or camelid binders or alternative formats can also be used as recognition domains [44]. The advantage of camelid sdFv is the reduced genetic load (half the size), reduced immunogenicity and the reduced tendency for aggregation while retaining the same specificity and affinity [45]. For hidden epitopes, the sdFv may be advantageous for the initial interaction of the targeted epitope compared to scFv based targeting due to less steric hinderance, higher solubility and the stability. Further, ligand-based CAR recognition domains have been introduced to target BCMA via trimeric APRIL [46], and the small chlorotoxin, a naturally derived 36-amino-acid-long peptide found in the venom of the death stalker scorpion leiurus quinquestriatus, which selectively binds to primary brain cancers is used for the treatment of glioblastoma (GBM) [47]. The basic requirement of recognition domains is the specific and rapid binding to the targeted antigen with the recognition domain to facilitate the CAR engagement. 

The structural domains including the spacer (also called hinge) and transmembrane domains stabilize the receptor and allow the functional presentation of the recognition domain. They shape the extracellular configuration of the receptor and connect the extracellular domains to the intracellular modules of the receptor to facilitate an efficient mechanistic signal transduction to the intracellular signaling domains. Various protein subunits derived from CD8a, CD28, and IgG hinge regions also in combination with IgG CH_2_ and CH_3_ domains and others have been utilized as spacer domains, which have shown distinct properties. The most frequently used transmembrane domains are derived from the CD8a and CD28 [48]. 

The intracellular signaling domains usually contain one or more costimulatory domains and a signaling domain. Costimulatory domains are mainly derived from two families, namely the immunoglobulin superfamily, which is represented by CD28 and ICOS, and the tumor necrosis factor receptor superfamily (TNFR) represented by 4-1BB, OX40 and CD27. Signaling domains are mainly derived from the CD3ζ chain, while alternative signaling domains such as DAP12 have been used [49,50,51]. 

## 4. Exponential Evolution in CAR T Cell Development

The early development of CAR receptors was hampered by the limited speed in molecular and synthetic biology in the late 1980s to perform high-throughput screenings [35]. The basic technologies required for CAR generation have evolved rapidly and made CAR manufacturing a standard GMP procedure [52] that can be partially automated today [53]. In the past decade, CAR patenting activity has exponentially increased by 100-fold from academic institutions and pharmaceutical companies [54], demonstrating the clinical and commercial impact of CAR T cell therapy today. Advancements in synthetic biology and gene synthesis technology has come to speed and allows screening of large gene libraries with thousands of different CAR constructs in a very short time nowadays, making the work much more time-efficient and studying detailed variations of CAR receptors possible. For instance, CAR receptor signaling can be systematically evaluated in response to combinations and mutations in costimulatory domains, transcriptional regulation enhancement and perturbation, gene knockdowns, knockouts, and knockins, which could not be addressed in the past in a timely manner [55,56]. Moreover, the refinement of phage display [57] and deimmunization strategies [58,59] have dawned a new era of generating binding sequences such as scFvs according to biological requirements at a high pace, compared to conventional laborious methods including mouse immunization followed by hybridoma screening, single-B-cell screening, and the use of transgenic mice with fully human variable regions to discover fully human mAbs through mouse immunization and screening [42,60].

## 5. The Evolution of CAR Receptors

**Figure 2:**
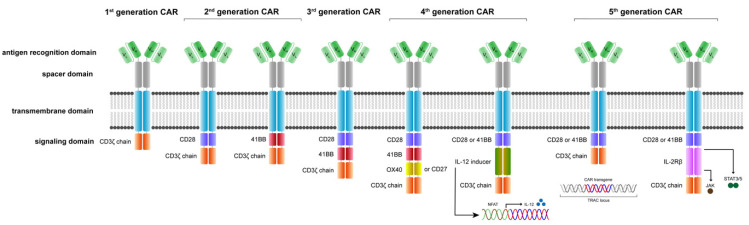
Evolution of CAR receptors.

The evolution of CAR T cells is illustrated in Figure 2. The original concept of a T body, considered as the prototype of a CAR, was invented by Eshhar et al. in the 1980s [35]; following that, the first scFv-based CARs, which were also created by Eshhar et al. in the early 1990s [38]. The critical step in the evolution of CAR T cells was the introduction of a costimulatory domain in the late 1990s by various CAR labs all around the world to mature from a first- to second-generation CAR [37]. From today’s perspective, first-generation CARs remain historic anecdotes. 

In the evolution of CAR design, the number of intracellular signaling domains were increased in later generations to enhance the potency and persistence of the CAR T cells. The extracellular domain is comprised of an antigen recognition domain, followed by the spacer, the transmembrane domain and the intracellular signaling domains. First-generation CAR T cells relied on the signaling of the CD3ζ chain only, whereas second-generation CARs incorporate two signaling domains, and third-generation CARs three signaling domains. The nomenclature of higher CAR T cell generations or next-generation CAR T cell technologies is not clearly defined. Fourth-generation CAR constructs may incorporate four signaling domains, may incorporate an inducible suicide switch (iCasp9) [61] or may conditionally secrete cytokines such as IL-12 in a CAR activation-dependent manner under an inducible promotor containing NFAT, NF_K_B or AP-1 responsive elements [62]. Site-specific CAR transgene integration at the TRAC locus leads to a functional collapse of the CD3 complex (TcR knockout, abrogation of GvHD) and may facilitate a more physiological CAR expression [63] and can be considered a fifth-generation CAR T technology. Additionally, the integration of IL-2Rß signaling that allows JAK/STAT pathway activation has been used under the term fifth-generation CAR technology [64]. Most used and validated costimulatory signaling domains include CD28, 4-1BB, OX40 and CD27 or a combination thereof. CD3ζ chain signaling is the most common signaling component of CAR receptors to date. CAR: chimeric antigen receptor. V_H_/V_L_: variable heavy chain and variable light chain of a single-chain variable fragment (scFv).

Although CAR technology has gone through a fast human-made scientific evolution, awareness of the rather slow progress in CAR technology shall change our language around CAR T cells being a novel kind of treatment. In recent years, high-throughput synthetic biology has come to speed and has led to a significant acceleration in the development of applied molecular genetics [55,56]. 

**Figure 3:**
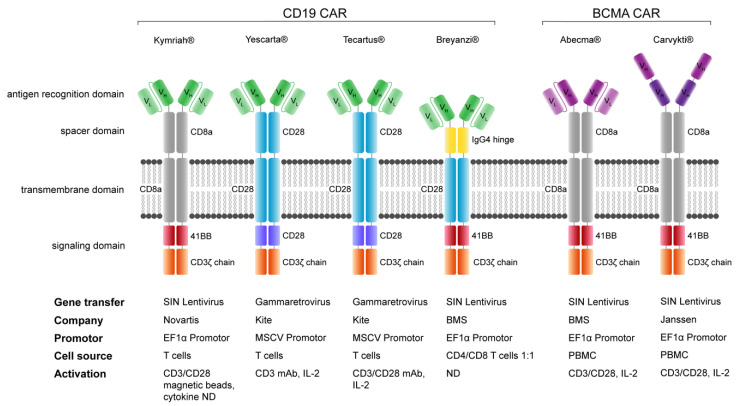
FDA-approved CAR T cell products.

The early first-generation CARs demonstrated limited activity due to various factors, largely attributable to failure in generating high-quality CAR products and the design of the molecular structure of CARs [38]. First-generation CARs comprised only a CD3ζ chain signaling domain, which lead to poor signal transduction, resulting in antigen-specific in vitro activation of CAR T cells that have cancer cell killing activity, but lack the ability for sufficient proliferation and engraftment in vivo [65]. Costimulatory domains derived from activating immune co-receptors such as the CD28 family and the TNF-receptor family are introduced in second-generation CARs [36,37], resulting in a sufficient signal transduction that leads to a stronger activation, cytokine production, proliferation, persistence and increased fitness of CAR-expressing effector T cells [36,37,65,66]. All currently FDA/EMA-approved CAR products are second-generation CAR-T cell products. They are illustrated in Figure 3 and will be discussed in a later section. Additional attempts to improve the potency of CAR constructs are illustrated in Figure 2. Yet, there are numerous other technologies that are not included in this review.

## 6. Link of CAR Architecture and Function

The CAR architecture and its modules define the function of CAR receptors. The overall performance of CAR-expressing cells is defined by the cell type and cell origin (NK cells [67] versus T cells [68]), and the immunophenotype representing the multiplicity of interacting immune receptors [69]. The subunits of a CAR are clearly correlated with their primary as well as secondary functions. Shaping CAR function is possible; however, complete control of CAR function by architecture design is impossible because artificial proteins have their own properties and always lack evolutionary-based optimization. 

The primary function and performance of a CAR are antigen recognition and engagement of the CAR-expressing cell with the target cell, the activation of the effector cell, polarization, formation of the cytolytic synapse, the initiation of cytotoxic action and induction of apoptosis in the target cell-mediated by the CAR, as well as the alteration of the gene expression and the persistent genetic imprint. The secondary function of CAR-expressing cells is more complex and more difficult to assess, simply for the factor of time. Long-term persistence, cellular metabolism, and its impact on cell fate are defined by non-immediate interactions and mechanisms. Most of the clinically relevant knowledge that we acquired about CAR-expressing cells is from applications in humans [70]. The translation from mice to human is the most challenging step. Curing mice is “easy” compared to curing humans. Mice are fantastic models to understand the biology of CARs, but real understanding is gained through human applications. As a result, despite accelerated development in preclinical sciences including gene synthesis, large high-throughput data acquisition and analysis technologies supported by artificial intelligence, only time-consuming clinical trials that take years to complete will reveal the truth of CARs in the context of human patients. Hence, clinical trials present the bottleneck in CAR development, especially in rare cancers with low patient numbers such as in pediatrics [70,71]. Nonetheless, all the excellent preclinical work provide the objectives and the rationale to run the most promising clinical trials and consequently save time in the development of next-generation CAR therapeutics. 

The basic and simplified principle of a CAR receptor is to make an effector cell, e.g., a T cell, specifically engage with a target cell that expresses the targeted antigen, for instance CD19. By recognizing and binding to the target antigen, the CAR-expressing cell is strongly attached in close proximity at approximately 10–40 nm distance to the target cell, a distance comparable to TcR–pMHC interactions [72,73]. The CAR receptor is constructed in a way that it transduces an activation signal into the CAR T cells, which in most cases mimics the response of a T cell receptor (TcR) via CD3ζ chain signaling. Basically, the mechanic lever of the CAR receptor leads to a signal transduction into the T cell, mimicking TcR signaling, which triggers a complex downstream signaling machinery with a multitude of effector functions within minutes [74]. Thus, a CAR is hijacking the function of the TcR to efficiently target surface expressed antigens in a MHC-independent non-restricted manner. However, CARs are not as good as canonical TcRs. Given the fact that TcRs are perfected by evolution over several hundred millions of years [75] and CAR technology has only a short history of development of 30 years [35,38], the results we have achieved using CARs are quite remarkable. On the other hand, failures in CAR development have taught us to appreciate the importance of the biology of effector cells, especially of T cells, and cancer biology in order to advance CAR therapeutics to the next level. This topic is discussed further in the review article by Waldman et al. “A guide to cancer immunotherapy: from T cell basic science to clinical practice” [76].

## 7. CD19—A Curse and Blessing

CAR T cell functions have been widely studied in CD19-CARs. So, why is that the case? The answer is shockingly simple—because CD19-CAR-Tcells work remarkably well. Several factors have facilitated successful treatment with CD19-CAR-T cells. 

### 7.1. CD19 Antigen

One crucial factor is the suitability of CD19 to serve as a CAR-targeted antigen [2,3]. The optimal cancer antigen is differentially overexpressed in cancer tissues and is not co-expressed on vitally essential tissues, and it is homogeneously expressed at high levels in all cancer cells [77]. Furthermore, it shows stable antigen expression irrespective of the cell cycle or treatment with no escape mechanism such as downregulation or loss of the antigen, and the antigen must be accessible for a CAR expressed by an effector cell [78,79] and not only by a soluble protein such as an antibody. On these premises, CD19 is not the perfect CAR antigen as patients treated with CD19-CAR-T cells develop immune escape variants. Nonetheless, CD19 is the best CAR target antigen available to date in terms of clinical efficacy [80]. 

CD19 is almost a perfect antigen with high expression levels in a large fraction of acute and chronic B-lineage-derived malignancies [81] with high and stable expression from early progenitor cells to late maturated B cells [77]. Besides, the generally high antigen density of CD19 in the range of several thousand molecules per cell (4000–25,000/cell) in BCP-ALL [82]. CD19 is a high-quality antigen because of the small size of its extracellular domain (271 amino acids), the configuration of the extracellular domain and the easy accessibility of its targeted epitope [83,84]. In multiply relapsed disease, patients may experience CD19_low_ (several hundred molecules per cell) or negative leukemia, yet the expression level in most cases still exceeds the expression of CD22, an alternative CAR-targeted antigen [83,84]. To recruit CAR-T cells to lyse target cells under optimized in vitro conditions, as low as 200 molecules per cell can be sufficient. However, to induce cytokine secretion, 10× more molecules are required [85] and the activation threshold clearly depends on the CAR architecture, especially the costimulatory domain and of course also on the targeted antigen [86]. 

High-affinity anti-CD19 antibodies have been generated by immunization of mice. CD19 carries several immunogenic epitopes, with one prominent epitope (around loop [87,88,89,90,91,92,93,94,95]), against which numerous high-affinity antibody clones (FMC63, AB1, B4, 4G7, HD37, BU12, F974A2, and SJ25) have been generated [96]. The most commonly used scFv in CD19-CAR-T cells is based on the murine FMC63 clone [26] which binds CD19 at a picomolar affinity (0.32 nM) [97]. Fortunately, the FMC63-based scFvs do not show any tendency for tonic signaling in the context of CAR-expressing cells [98]. All FDA/EMA-approved CD19-CAR-T products are based on the murine FMC63 recognition domain (scFv) [26].

### 7.2. CD19 in Comparison to Other Leukemia-Associated Antigens

The distance of the T cell to the target cell is critical for optimal effector function. In native T cells, the TcR–pMHC interaction occurs at a distance of approximately 15 nm [99,100]. For optimal CAR T cell engagement, the distance between the CAR T cell and the target cell is also a determining factor of CAR function [101]. CD19 appears to be an optimal CAR target antigen compared to other interesting alternatives such as CD22 [102].

The spatial distance of T cells engaging with virus-infected or cancerous cells via T cell receptor (TcR) engagement is approximately 15 nm. Most CAR receptors mimic the function of the TcR via CD3ζ chain signaling. FDA/EMA-approved CAR products are optimized to operate at the distance of TcR–MHC synapses. If the distance between the effector cell and the target cell is too long, the formation of the cytolytic synapse is impaired and CAR targeting is non-efficient which results in poor CAR function. Thus, choosing the best suitable targeted epitope is critical for the function of CAR T cells targeted to proteins with a large extracellular domain. However, target antigens cannot be modified and therefore the CAR receptor must be adapted perfectly to engage with the target antigen and initiate the formation of the cytolytic synapse. CAR function in CD19 and BCMA-targeting CARs is supported by the small extracellular domain. CD22 CARs require targeting of a proximal epitope, since targeting a distal epitope of the large extracellular domain hinders the formation of a cytolytic synapse [103,104]. The proximal epitope is recognized by the mouse antibody clone m971. More distal epitopes are targeted by the mouse antibody clones HA22 and BL2 which do not translate in any relevant effector function used in CAR-expressing cells [104]. In the FDA/EMA-approved CAR-T cell products, the recognition domain for CD19-CAR-T cells is based on a scFv, derived from the mouse anti-CD19 antibody clone FMC63 [21], and in idecabtagene vicleucel Abecma^®^, the only BCMA-CAR-T cell product on the market to date, was constructed from the mouse anti-BCMA antibody clone C11D5.3. 

Alternative target antigens in the treatment of BCP-ALL are CD20, CD22, CD38 and CD79B. The expression level varies significantly between CD10^+^ and CD10^−^ BCP ALL, with unfavorable prognosis of CD10^−^ leukemia [105]. CD38 is homogenously expressed across BCP-ALL, whereas CD22 has a higher expression in CD10^+^ BCP-ALL, and CD20 is expressed only in CD10^+^ in 42% of patients [106].

**Figure 4:**
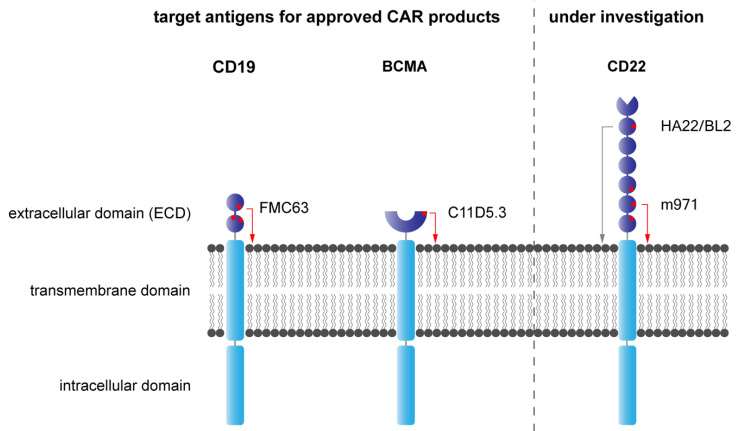
Mechanistic challenges of CAR-targeted antigens.

In patients who experienced CD19-negative relapse after CD19-CAR-T cell treatment, CD22-targeted CAR T cells are able to induce complete remissions [107]. However, the expression of CD22 in general is lower than CD19 and leukemia-free survival is significantly lower than in CD19-CAR-T cell therapy [104]. Due to the high risk of relapse post CD22-CAR-T cell therapy, subsequent allogeneic stem cell transplantation in molecular remission is highly recommended as patients are unlikely to survive without consolidation therapy by allo-HSCT [84]. Relapsed patients showed a significantly reduced CD22 expression at diagnosis compared to the pre-treatment condition, which is indicative of the selective evolutionary pressure. Thus, combinatorial CD19-CD22 bivalent CAR T cells may reduce the risk for leukemia recurrence and are studied in clinical trials in children and adults [80]. In preclinical models, trispecific CD19-CD20-CD22 CAR T cells can control heterogenous cancers [108,109]; however, antigen loss remains the major cause of CAR T cell resistance also in dual-targeted CAR therapies [80]. Strategies to specifically increase the target antigen expression by co-administration of medications, such as Bryostatin1 to increase CD22 levels, can improve CAR T cell performance, but curing patients will depend on a robust target antigen expression [110]. In preclinical models, CD20 [111], CD22 [84], CD38 [112] and CD79B [87] CAR T cells have been proven efficacious and are used in clinical trials to treat B-lineage malignancies. CD38 CAR T cells can also be used for the treatment of T ALL and AML [113], but they are associated with a broader spectrum of toxicities in the lymphoid and myeloid compartment and leads to fratricide of early T cell progenitor cells [112]. In Figure 4, the structural properties of the CAR target antigens CD19, BCMA and CD22 are illustrated.

The use of CAR T cells for the treatment of acute myeloid leukemia (AML) is challenging due to dramatic on-target off-tumor toxicities. The most effective AML-associated CAR-targeted antigens such as CD33 and CD123 are co-expressed in hematopoietic progenitor cells [114]. Strong activity of CD33- [115] or CD123-CAR-T cells can lead to profound depletion of the myeloid compartment [116,117] that is acceptable only for a limited time frame within the range of several weeks because lethal infectious complications including systemic and invasive bacterial and fungal infections result from mid-term agranulocytosis, which is a major cause for transplant-related mortality in allogeneic stem cell transplantation with delayed myeloid immune reconstitution [118]. Many cancer-associated overexpressed antigens cannot be targeted continuously on a tissue-depletion level, because lethal inflammatory complications can lead to organ failure. Thus, transient targeting may provide a solution for targeting non-exclusive overexpressed antigens as CAR targets.

## 8. A Paradigm without a Shift—Affinity and CAR Performance

In the context of CAR-mediated cancer targeting, predominantly the affinity of the recognition domain, but also the signaling as well as the structural domains determine the CAR activation threshold to the corresponding target antigen density, which allows the CAR to successfully recognize and engage with low antigen-expressing cancer cells [86,119,120]. As immune escape mediated by downregulation or any functional antigen loss is the major cause of relapse in CD19-CAR-T cell-treated patients, it appears favorable for CD19-CAR-T cells to also engage with CD19_low_-expressing cancer cells [2,78,121] at the price of on-target off-tumor toxicity on healthy cells, e.g., neurons with low CD19 expression. High-affinity CD19-CAR-T cells (FMC63, K_D_ 0.32 nM) may have a lower risk of antigen escape variants as a result of reduced CD19 antigen expression required to be recognized and eliminated by high-affinity CD19-CAR-T cells, compared to moderately reduced-affinity CD19-CAR-T cells (CAT19) [2,97]. Conversely, the severe neurotoxicity (ICANS) can be ameliorated by using reduced-affinity CD19-CAR-T cells (CAT19, K_D_ 14 nM), which may however not engage with CD19_low_-expressing cancer cells on the other end. Despite that CAT19 CD19-CAR-T cells have been reported to show a greater tendency for rapid expansion and persistence compared to high-affinity CD19-CAR-T cells based on (FMC63), the commonly observed life-threatening toxicities CRS and ICANS occurred significantly less [97]. However, comparing the outcome of high-affinity versus reduced-affinity CD19-CAR-T cell-treated patients, event-free survival revealed substantial differences. Under the current circumstances with limited clinical data available, the most relevant and alarming discriminator of high- versus reduced-affinity CD19-CAR-T cell-treated patients seems to be the significantly increased risk of CD19-negative and CD19_low_ relapse in the reduced-affinity CAT19 CD19-CAR-T-treated cohort (35%, 5/14 pts) [97] compared to 10–20% in high-affinity FMC63-based CD19-CAR-T-treated patients [2]. With regard to mediating tumor lysis via secondary mechanisms, cytokine secretion plays an important role to facilitate the elimination of antigen_low_ and antigen-negative tumor cell populations [122], and thus activation thresholds based on the variables antigen density, scFv affinity and signaling also determine the performance of CAR T cells in antigen-negative cancer cells [30,86].

### 8.1. Comparison of the T Cell Receptor and a Chimeric Antigen Receptor

The recognition domain is one of the subunits responsible for determining the antigen-density activation threshold in CAR-expressing immune cells [101]. The signaling domains also have an impact on the antigen-density threshold [86]. As discussed above, in comparison to TcRs, the activation threshold of CAR receptors is at least a 100-fold higher [123]. This means 100-fold more antigens on the cell surface are necessary to engage a CAR T cell compared to native unmodified T cells via TcR engagement [124]. Even though TcRs have an affinity in the micromolar range (1 to 50 uM), the signaling, especially the ZAP70 recruitment of the CD3ζ chain, is much more efficient in native TcRs compared to artificial CAR receptors [125]. In general, TcR-mediated and CAR-mediated targeting involve two separate mechanisms with the same goal to eliminate aberrant cells. While TcRs are highly specific to changes in the genome, e.g., mutations or foreign non-human proteins, CARs recognize cancer-associated antigens. The level of aberrant peptides presented by MHC (pMHC) in cancer is very low [126], inconsistent and heterogenous compared to pMHC in virus-infected cells [127]. In virus-infected cells, the interaction of TcR and MHC is more potent and still the total number of available pMHC complexes often is lower than the minimum target antigen expression required for substantial CAR engagement [84,86,127]. These comparisons are important and drives major implications on how to reconstruct new artificial receptors with higher sensitivity [123,128]. Despite TcRs having the superior capability for lower target antigen density, CAR-expressing cells are extraordinary in their performance, as CAR receptors facilitate antigen-specific immune responses in a novel mode of action that native T cells with their TcRs cannot achieve. CAR signaling must be adapted to the expressed antigen density in wise consideration of the targeted antigen to balance efficacy and toxicity [129,130]. Therefore, the super high sensitivity of TcR-mediated targeting for the target antigen CD19 would potentially lead to self-destruction of vitally essential tissues including CD19^+^ cells in the CNS. High-sensitivity CAR designs may also drive exhaustion due to the abundance and consecutive overactivation during the targeting process [129]. T cells, unlike CAR-T cells, are designed to detect and function on a low or very low-antigen pMHC frequency level [123]. In general, depending on the targeted antigen, CAR function requires adaptation to the expression level of the targeted antigen. As there are no exclusive surface expressed cancer antigens (MHC independent), the sensitivity of the CAR has to be fine-tuned and adapted to react robustly with cancer cells and shall not engage with healthy tissue at best, if the target antigen is co-expressed on vitally essential tissues [97,119]. In some respects, the almost exclusive target antigens CD19, CD20 and CD22 for B-lineage-derived cells are exceptions, as the B-lineage compartment can be regarded as non-vitally essential tissue [131]. CAR receptors by nature cover a different range of target antigen density than TcRs and to date the activation threshold has not been successfully tuned to the same sensitivity level as TcRs [123]. The clou of CAR-mediated targeting is not to find a way to make a CAR another variant of a TcR, which it is not, but rather to appreciate the limitations of CAR targeting and identify smart combinations of CAR targeting to increase the potency of CAR technologies [80,132]. 

### 8.2. Requirement for CAR Optimization per Antigen

For every target antigen, the challenge is to identify the best CAR architecture with an optimized sensitivity for that particular target antigen to balance anticancer activity and on-target off-tumor toxicity in order to prevent fatal complications [130]. The threshold is thought to be above the low threshold target antigen density of CD19 [129]. To date, no CAR T has been identified to target at a lower antigen threshold density (molecules per cell surface) than CD19-CARs and most likely the target antigen threshold for most antigens will be in the range of several 1000 molecules per cell and above [30,84,86]. Understanding the complexity of CAR targeting requires rethinking and thinking beyond CD19. Even though CD20, CD22 and BCMA are almost exclusive B-lineage-derived antigens, the targeting is less potent and reveal the molecular challenge in CAR T cell therapy, which are the identification and fine-tuning of the most efficient CAR T cells in an approach tailored to the target antigen expression. It is noteworthy that high-affinity CARs compared to low-affinity CARs show an improved recognition capability of low-expressed target antigens. On the other hand, they are more likely to exhaust, and long-term persistence may be impaired. Further, CRS and ICANS are more common in high-affinity CD19-CAR-Tcells [97]. As a result, patients who receive low-affinity CD19-CAR-T cell treatment appear to be at a higher risk to experience CD19_low_ and CD19-negative leukemia recurrence which is less common in high-affinity CD19-CAR-T cell therapy [2,97]. 

Generally speaking, the sweet spot of CAR targeting is reached at the point where cancer is specifically targeted, while the CARs are not overactivated, lose their fitness through exhaustion and lack persistence. There is a need to achieve a target antigen-specific balance to facilitate robust anticancer immunity with acceptable on-target off-tumor toxicity [119]. Current CAR concepts are limited in their ability to meet these complex and dynamic criteria, but next-generation CAR designs with the ability of combinatorial targeting may solve some of these problems [132]. 

## 9. Immunogenicity of CAR Products

Originally, CAR receptors used to be artificial immune receptors composed of murine and human protein sequences making them a chimeric receptor. Today, fully human CAR receptors can be generated [133], which should be appreciated in the nomenclature of artificial immune receptors. This may seem to be a minor difference in the molecular anatomy and evolution of CAR receptors, considering the few changes of the amino acid residues only in the recognition domain, the single-chain variable fragment (scFv) [134].

However, these minor differences in immunogenicity may as well be one of the key changes, making CAR-expressing cells applicable to a broader range of antigens with reduced risk of antibody-mediated CAR rejection [135]. Another way of potentially decreasing the immunogenicity of a recognition domain is to reduce the size and simplify the structure to single-domain heavy-chain-only binding domains [136]. By CAR-specific depletion of the antibody producing cells including the B cells and the plasma cells, CAR effector cells targeting B-lineage malignancies inherently suppress the generation of anti-CAR antibodies. However, antitransgene rejection of CAR T cells has been observed in CD19- and CD20-CAR-T cells [137] as well as in CAR-T cells targeted to non-B-lineage-associated antigens^131^. Thus, the function of the B-lineage compartment is dramatically impaired by CAR T cell therapy targeted to B-lineage-associated antigens such as CD19, CD20, CD22 and BCMA [138,139]. 

Anti-CAR humoral response is capable of rejecting non-human proteins, especially those highly expressed and accessible on the cell surface such as CAR receptors on CAR-expressing cells distributed in the whole body [103]. Besides the production of immunoglobulins, B-lineage-derived cells are regarded as professional antigen-presenting cells (APCs) to ensure effective production of high-affinity antigen-specific antibodies while minimizing the production of non-specific antibodies and auto-antibodies [140]. As CAR receptors are expressed at very high levels > 50,000 molecules per cell [141], presentation of non-human immunogenic peptide sequences by MHC bears the risk of T cell-mediated immune rejection. Thus, the risk of T-cell-mediated CAR elimination is also reduced by depleting B-lineage-derived cells which act as antigen-presenting cells.

Targeting non-B-lineage-associated antigens does not impact on the B-cell compartment and thus does not inhibit its function. Logically, both the risk of immune rejection of CAR-expressing cells via antibodies targeted to the murine extracellular component of the CAR as well as T cell-mediated CAR rejection are higher in CAR T cells targeting antigens that do not suppress the humoral immune response. 

Potent strategies to reduce the risk of immune-mediated rejection of CAR-expressing cells include deimmunization, humanization and the generation of fully human CAR sequences [58,133,142]. In theory, fully human CAR constructs should not be recognized as foreign proteins and trigger an immune response. The truth, however, is that immune rejection may occur in response to any synthetic protein as it is of non-human origin and in the light of autoimmune phenomena, we know that naturally present physiological human proteins may be attacked by the immune system as a result of cross-reactivity with immune responses against pathogens (virus, bacteria, fungus) [143]. Errors in the maturation of immune cells may cause transient or chronic autoinflammation, partially leading to devastating autoimmune diseases such as Crohn’s disease [144,145]. The development of antidrug antibodies against the fully human anti-TNFα antibody adalimumab is associated with treatment failure [146]. Nonetheless, all mentioned strategies to reduce the immunogenicity of foreign proteins have been proven efficacious. 

## 10. Comparison of FDA/EMA-Approved CAR-T Cell Products

The CD19-targeting FDA/EMA-approved CAR-T cell products are constructed in distinct architectures, even though they share the same recognition domain derived from the murine FMC63 IgG2a antibody clone with the same orientation (VL-VH) of the single-chain variable fragment (scFv) [21]. Despite these differences, all CD19-CAR-T cell constructs—tisagenlecleucel (marketing name Kymriah^®^), axicabtagene ciloleucel (marketing name Yescarta^®^), brexucabtagene autoleucel (marketing name Tecartus^®^), and lisocabtagene maraleucel (marketing name Breyanzi^®^)—have demonstrated outstanding clinical performance in various B-lineage malignancies [2,3,22,23,24,147,148]. Tisagenlecleucel was FDA approved and later approved by the EMA based on the findings in the clinical trial “Study of Efficacy and Safety of CTL019 in Pediatric ALL Patients (ELIANA)” with the ClinicalTrials.gov identifier: NCT02435849, funded by Novartis [2,21,149]. Recently, the BCMA-targeted CAR T cell product idecabtagene vicleucel (marketing name Abecma^®^) was US-FDA approved for the treatment of multiple myeloma with identical molecular architecture to the CD19-CAR T cell product tisagenlecleucel [21,25,150].

Currently, six different CAR T cell products are approved by the US-FDA and/or the EMA for the treatment of refractory patients with B-lineage-derived cancers including ALL, lymphomas, and multiple myeloma. All products are based on a second-generation CAR architecture with one costimulatory domain and CD3ζ as the signaling domain. Interestingly, the CD19-CAR-T cell products use different spacer, transmembrane and costimulatory domains. Kymriah^®^ is approved for use in pediatric patients and young adults (<25 years). The integrated table provides details on the gene transfer, the marketing company, the constitutive promotor, the cell source and information about the activation and culturing conditions if accessible. VH/L: heavy/Light chain of single-chain variable fragment (scFv). SIN Lentivirus: Self-inactivating Lentivirus. PBMC: peripheral blood mononuclear cell. ND: not disclosed.

Evidently, CD19 is a perfectly well-suited CAR target antigen with different CAR constructs being efficacious for patient treatments [26,78,151]. This is not the case for most CAR-targeted antigens, which prove to be more challenging for various biological reasons, including the expression level [83] and expression in cancerous tissue as well as vitally essential tissues, the size of the extracellular domain [102], configuration and accessibility (hidden epitopes) of the targeted antigen [84,104,152]. The most obvious difference of the CD19 CAR constructs lies in the costimulatory domains 4-1BB or CD28 which lead to a differential gene expression signature of >200 genes [153], despite the shared bidirectional activation of the NF-kB and mTOR pathway with the induction of proinflammatory cytokine production such as IL-2 and IL-6 as well as the expression and activation of antiapoptotic proteins such as BCL-xL [154,155,156]. The biological consequences of CD28 and/or 4-1BB costimulation are diverse, with distinct differences in response kinetics, cell cycling, clonal expansion, survival, metabolism, and long-term persistence in vitro and in vivo.

Depending on the requirements of the CAR, the features of CD28 or 4-1BB costimulation may be advantageous [157]. Costimulation by a receptor of the TNFR family such as 4-1BB leads to increased oxidative metabolism, mitochondrial biogenesis and mitochondrial fitness and capacity associated with the pronounced maturation in central memory T cells with enhanced persistence, whereas costimulation by the CD28 family leads to increased glycolytic metabolism, reduced mitochondrial biogenesis, fitness and capacity associated with the maturation to effector memory T cells with shortened persistence [158]. The strongest activating costimulatory domain is CD28. Clinically, CD28 costimulation leads to a more rapid expansion of the CAR T cells accompanied by life-threatening adverse events such as cytokine release syndrome (CRS) and immune effector cell-associated neurotoxicity syndrome (ICANS) [159]. Preclinical studies have revealed that CD28-costimulated CAR T cells express higher levels of exhaustion markers such as PD-1, TIM3 and LAG3 compared to 4-1BB-costimulated CAR T cells [120,160]. To date, there are no sufficient biological data available in a clinical setting to allow a conclusive comparative analysis of CD28 versus 4-1BB costimulation in CAR T cells. Despite the unclear data landscape in preclinical models with regard to enhanced persistence of CAR T cells, most likely due to the short observation time of less than 3 months in most mouse studies [157,161], greater persistence in 4-1BB-costimulated CD19-CAR T compared to CD28-costimulated cells were observed in various clinical trials and are in general accepted, even though the value of CD19-CAR persistence remains elusive and seems to be cancer-specific [2,3,162]. In adult patients with B-NHL lymphomas and B-ALL, CD19-CAR T long-term persistence does not correlate with response to treatment and long-term cancer-free survival [163], whereas in pediatric ALL patients, persistence for over 6 months appears to be the determining factor for long-term leukemia clearance or leukemia recurrence in case of shorter CAR persistence [2]. Thus, in pediatric and adolescent patients, 4-1BB costimulation in CD19-CAR-T cell therapy may be superior compared to CD28 costimulation for the treatment of B-cell precursor ALL.

## 11. State-of-the-Art CAR T Cell Therapy in Children

Outcome of relapsed and refractory BCP-ALL remains poor at approximately 40% with a median survival of 14 months despite the use of allogeneic hematopoietic stem cell transplantation and the emergence of novel therapies in recent years [164,165,166]. CD19-targeted therapies including the bispecific T cell engager (BiTE) therapy blinatumomab and the even more potent CD19-CAR-T cell therapy have been proven efficacious in heavily pre-treated patients, albeit with severe but widely accepted toxicities due to the lack of alternative treatment options [2,167]. As outlined above, the main reason why CD19 qualifies for highly potent and long-term immunotherapy is the differential overexpression of CD19 on malignant blasts compared to the low expression levels on vitally essential tissues, such as low-level expression on neural tissues [168,169]. Tisagenlecleucel therapy provides cures to patients who were considered incurable until CD19-CAR-T cells were used in a substantial number of patients and continuously showed high complete remission induction rates and durable leukemia-free survival [2,17,78,149].

### 11.1. Clinical Indication for CD19-CAR-T Cell Product Tisagenlecleucel (CTL019, Kymriah^®^)

The indication for treatment with tisagenlecleucel in children and young adults (3 to 25 years) is relapsed or refractory pediatric B-cell ALL. Tisagenlecleucel treatment is approved for autologous CAR T cell therapy only. The major eligibility criteria include the presence of >5% blasts at screening, second or subsequent bone marrow relapse, or bone marrow relapse after allogeneic hematopoietic stem cell transplantation and must be ≥6 months from HSCT. The definition of refractory is not achieving an initial complete remission after two cycles of standard chemotherapy regimen (primary refractory) [21,149].

### 11.2. Tisagenlecleucel Therapy

**Figure 5:**
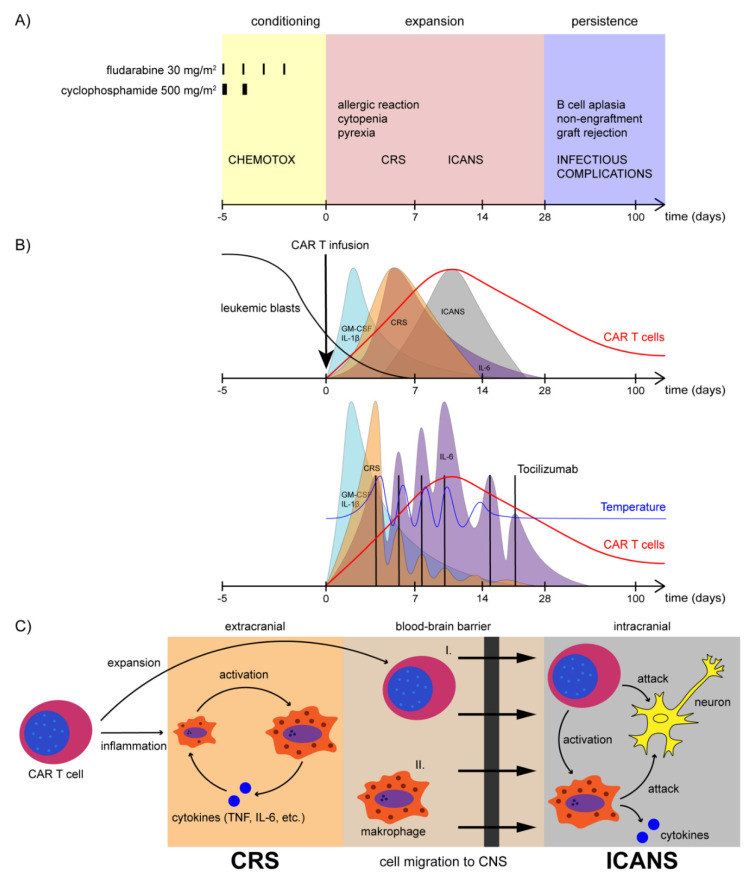
CAR T cell treatment and complications.

According to inclusion and exclusion criteria, eligible patients are identified and required to undergo unstimulated mononuclear cell apheresis. Subsequently, the apheresis products are evaluated first for manufacturability, before the patient is approved eligible for tisagenlecleucel treatment. In the meantime, patients receive an individual bridging therapy according to the treating physician [21]. An overview of treatment with CAR T cells including the lymphodepletion, the most common adverse events and the pathophysiology of CRS and ICANS is illustrated in Figure 5.

After clearance of the patient-individual CD19-CAR-T cell product tisagenlecleucel, the patient undergoes a preparative lymphodepleting chemotherapy. Lymphodepletion includes 4 doses of fludarabine (Flu) at 30 mg/m^2^ and 2 doses of cyclophosphamide (Cy) at 500 mg/m^2^. Lymphodepletion paves the way for CAR T cell engraftment by eradication of immunosuppressive cells such as regulatory T cells (T_REG_) and myeloid-derived suppressor cells (MDSCs), which leads to enhanced expression of costimulatory ligands on cancer cells, reduced elimination of relevant T cell homeostatic cytokine levels such as (IL-2, IL-7 and IL-15) thus promoting the initial anticancer immune response, exponential proliferation, robust engraftment and persistence of CAR T cells [170]. This may also induce immune tolerance and prevent rejection of chimeric transgene cells. Tisagenlecleucel may be infused into the patient from 2 to 14 days after completion of the (Flu/Cy) non-myeloablative lymphodepletion. CAR T cells are infused at 0.2 to 5.0 × 10^6^ tisagenlecleucel transduced viable T cells per kg body weight for patients ≤ 50 kg, or 0.1 to 2.5 × 10^8^ tisagenlecleucel transduced viable T cells for patients > 50 kg) [21,149].

It is noteworthy that a significant proportion of pediatric BCP-ALL patients do not survive while waiting for the production and preparation of CAR T cell products. In the ELIANA trial NCT02435849, a total of 107 patients were screened, 92 were enrolled, but only 75 (70%) underwent infusion [149] which meant 32 (30%) patients did not receive the CAR T cell treatment. Multiple factors contributed to the significantly reduced number of patients who finally received the tisagenlecleucel (CTL019) product including biological reasons, but also infrastructural reasons and time, which has a determining role in some patients’ lives. Centralized versus decentralized manufacturing is an ongoing discussion in the field. Decentralized on-time manufacturing may shorten the waiting time for the CAR T cell product and may reduce costs [171]. On the other hand, implementing tisagenlecleucel CAR T cell therapy earlier in the treatment algorithm will improve the outcome of CAR T cell therapy [172]. Hence, the success of CD19-CAR-T cell therapy using tisagenlecleucel was significantly lower when all enrolled patients (intent-to-treat) were taken into account, compared to exclusively analyzing patients who received the CAR T cell product. In the clinical trial NCT02028455, the decentralized CAR manufacturing improved the intent-to-treat to >90% [173] compared to 70% in the ELIANA trial [149]. Notably, the real-world outcomes for tisagenlecleucel showed the same efficacy and even a higher safety profile than in the pivotal study [174]. First presented data of brexucabtagene autoleucel by Wayne et al. in pediatric patients also demonstrated a reliable remission induction rate and an impressive leukapheresis to product release time of 14 days. Not unexpectedly, higher grades of CRS were observed (≥3 adverse events in 100% of patients); and among responders, CAR T cells were undetectable by 3 months post infusion [175]. Even though there is still a need for major improvements, it is beyond question that CD19-CAR-T cells and tisagenlecleucel especially are novel therapeutics that have contributed significantly to better outcome and prolonged survival in r/r pediatric BCP-ALL patients.

### 11.3. Follow-Up Patient Care Post Tisagenlecleucel Infusion

Usually, patients are closely monitored after tisagenlecleucel infusion for the first 28 days [21]. The follow-up intervals are extended in due course comparable to follow-up intervals common in autologous HSCT. However, patients are required to receive regular immunoglobulin replacement in case of sustained tisagenlecleucel persistence and consecutive B-cell aplasia that can cause chronic hypogammaglobulinemia-dependent humoral immune deficiency [131]. 

### 11.4. Allogeneic HSCT versus CD19-CAR-T Cell Therapy

It is difficult to make a direct comparison of clinical efficacy between allogeneic HSCT and CD19-CAR-T cell therapy, as HSCT improves outcomes in specific ALL populations, while CD19-CAR-T cell therapy has demonstrated efficacy in patients who failed allogeneic HSCT and/or are not eligible for HSCT [176]. Tisagenlecleucel CAR T cells exhibit impressive antitumor efficacy with superior complete remission induction of 81–90% and overall survival rates of 67–76% at 12 months, superior to conventional chemotherapy or HSCT [2,149,177]. In patients with high CD19 expression and no escape variants, CD19-CAR-T cell therapy is associated with less toxicity and superior leukemia-free survival than after HSCT. It is noteworthy that the combination of blinatumomab and HSCT also results in excellent survival rates in patients with complete MRD response [178]. Patients who show a tendency to develop CD19-negative tumor cell populations, e.g., during blinatumomab treatment, are likely to fail CD19-CAR-T cell therapy as well [2]. CD19-CAR-T cells cannot provide durable remissions for patients with CD19-negative cancer. In this case or if CD19-CAR-T cells do not persist > 6 months after infusion in BCP-ALL (but not for DLBCL), consolidative allogeneic HSCT may be a valid strategy to improve the outcome at the price of chemotoxicity from the conditioning regimen [2,179] and posttransplant complications such as acute and chronic GvHD as well as infectious complications [180]. Further, CD19-negative relapse post tisagenlecleucel occurs in 10–20% of patients [2]. Despite limited data available to date, CD22-CAR-T cell therapy is an option for patients who failed CD19-CAR-T cell therapy, with a high complete remission (MRD^−^) induction rate (61%). Yet, patients who do not undergo a consolidative allogeneic HSCT seem to be at very high risk of relapse [84,181]. Thus, CAR T cell therapy may be used for remission induction therapy in these patients and allow patients to proceed for an allogeneic HSCT in complete MRD-negative remission that cannot be achieved by any other treatment in chemorefractory patients for consolidation therapy [176,179]. Bivalent CAR T cell technologies address antigen escape but have not proven to solve the antigen question rigorously [80].

### 11.5. Cytokine Release Syndrome

In early-phase post CAR T cell administration, most patients develop an immune reaction with unspecific clinical symptoms such as fever, rigors, malaise, and anorexia [182]. Fever can reach high grades for a week or longer and may be accompanied by multiorgan dysfunction including dyspnea, lung edema, hepatic, and renal dysfunction as well as heart dysfunction, which can consequently lead to a life-threatening clinical state with multiorgan failure and death [183,184]. Today, cytokine release syndrome (CRS) can be treated successfully if CRS is detected at an early stage and the specific anti-inflammatory treatment is initiated without delay utilizing tocilizumab to block the IL-6 signaling and corticosteroids to dampen the overall immune response and dampen the CAR T cell function [182,185]. 

The conditioning regimen fosters the increased production and secretion of the TH1-associated cytokines IL-2, IL-7 and IL-15 after CAR T cell engagement with targeted antigen-positive cells. In stage one (1), the highly activated CAR T cells lyse target cells, secrete cytokines, and undergo polyclonal exponential proliferation. The cytokines promote survival and ongoing proliferation of the CAR T cells, and in parallel co-activate monocytes and macrophages which are capable of producing massive amounts of cytokines in any tissue. The systemic activation and secretion of cytokines by CAR T cells as well as monocytes and macrophages drive local CRS to become a systemic CRS. Thus, the second stage (2) of CRS is introduced by a second wave of cytokines which is predominantly characterized by high serum levels of GM-CSF and the pleiotropic cytokine IL-6, an early detection marker of CRS. In the third stage (3), CRS can evolve dynamically into a life-threatening cytokine storm based on the autocrine, paracrine and systemically paracrine pyramid activation system [186]. CRS may develop over a couple of days but may be initiated immediately after CAR T cell administration. Secondary, life-threatening neurotoxic complications usually develop during the course of CRS, but may also develop in patients with mild CRS or in patients with absent CRS [187]. The clinically predominant features of CRS may depend on the systemic involvement of different body compartments and organs [184]. The higher the grade of inflammation, the more severe the specific immune response. The secondary recruitment of accessory cells including monocytes, macrophages and endothelial cells further exacerbates CRS, leading to the increased risk of direct organ toxicity [186]. In the beginning of CRS, the brain is protected from primary and secondary involvement of CRS by the blood–brain barrier. The migration of CAR T cells to the brain is slower compared to other compartments of the body. In due course, the endothelial cells of the omnipresent vascular system contribute to CRS complications by expressing Ang-2 and *von Willebrand factor* triggered by IL-1 and IL-6 [188]. This makes the blood–brain barrier porous, allowing cytokines to intrude the central nervous system and affect the brain with increasing concentrations [186]. There is evidence that higher CAR T cell numbers and higher cytokine levels in the CNS promote ICANS. Yet, the complex pathophysiology of ICANS make it difficult to discern the severity of ICANS with simple measures such as cell numbers or with cytokine levels. Nonetheless, there is a clear correlation between the incidence of neurotoxicity and CRS [189]. In general, ICANS may be regarded as a local CRS of the brain. The main reason why the treatment and pre-emptive treatment utilizing the IL-6 receptor blocking antibody tocilizumab significantly reduces the risk for extracranial CRS, but not intracranial CRS (ICANS) is the lack of ability to reach sufficient blocking concentrations in the brain (approximately 1% of peripheral extracranial concentration) [190]. The clinical grading and management of CRS are well described in the article “Current approaches in the grading and management of cytokine release syndrome after chimeric antigen receptor T-cell therapy” [191].

### 11.6. Neurotoxicity

A range of neurologic symptoms after CAR T cell administration including headache, tremor, speech impairment, confusion, delirium, and reduced consciousness (lethargy, stupor, obtundation) are in the scope of the clinical presentation of ICANS [28]. B-ALL patients are at higher risk than lymphoma patients to develop ICANS even though the exact mechanisms for the development of severe ICANS are not well understood and severe CRS is one of the main risk factors for ICANS [28,192]. 

CD19 is a B-cell receptor (BCR) co-receptor almost exclusively expressed in the B-lineage compartment. Even though there is low expression of CD19 on neural tissues [168], the actual clinically relevant on-target off-tumor toxicity in neural tissue is limited to the very early phase of the treatment after infusion of the cells. ICANS usually develops on day 4–6 post CAR infusion and lasts for up to 14 days [193] during the exponential proliferation phase of highly activated CD19-CAR-T cells. It is the main cause of life-threatening events and fatalities especially in CD19-CAR-T cell treatments with CD28 as costimulatory domain [188,194]. There is an association between serum concentrations of IL-15, a key cytokine for T cell expansion and survival, and the development of ICANS [28]. Neurotoxicity due to cerebral edema lead to the termination of the phase II ROCKET JCAR015 CD19-CAR-T cell trial treating adult patients with r/r ALL [195]. The mechanism of neurotoxicity caused by CD19-CAR-T cell therapy is not fully understood and various factors appear to impact on the susceptibility and severity such as bone marrow disease burden, the use of cyclophosphamide and fludarabine for lymphodepletion, and the presence of any pre-existing neurologic comorbidity [187,188,194]. 

### 11.7. Macrophage-Associated Hyperinflammation

There are several distinct life-threatening inflammatory syndromes associated with macrophage-derived pathological hyperinflammation characterized by high persistent fever, cytopenia, liver dysfunction with coagulopathy accompanied by high cytokine levels (IL-1β, IL-6, IL-18, TNF, and IFNγ), hypertriglyceridaemia and hyperferritinaemia [196]. These are hemophagocytic lymphohistiocytosis (HLH) and macrophage activation syndrome (MAS) mostly triggered by viral infections, rheumatological diseases and inherited lymphoid immune cell dysregulation [197,198,199] or a gain-of-function mutation in the inflammasome component gene NLRC4 [200]; and in the era of T cell immunotherapies, HLH and MAS are also initiated via BiTE [201] and CAR T cell therapy [181].

A biphasic inflammatory response was observed after CD22-CAR-T cell therapy, with a self-resolving initial CRS and signs of HLH features. In a second wave, driven by a secondary CAR T cell expansion, patients developed HLH/MAS-like symptoms dissociated from the first CRS phase [181]. Morris et al. suggested a unique pathophysiology [202] but the clinical pattern may as well be a recurrence of the initial CRS with a triggered HLH/MAS component induced by the very same mechanism.

Major complications following CAR T cell therapy over the course of 180 days after infusion. (Figure 5A) Different phases of treatment with CAR T cells: conditioning (yellow) with lymphodepleting chemotherapy starts with 4 doses of fludarabine 30 mg/m^2^ and two doses of cyclophosphamide 500 mg/m^2^. CAR T cells are administered on day 0 and go through an expansion phase (red). After clearing the tumor cells, the CAR T cells enter the persistence phase (blue). Major complications are CRS and ICANS, both manifesting in the first days after treatment with CAR T cells. (Figure 5B) Kinetics of leukemic blasts (black) and CAR T cells (red) after conditioning and infusion of CAR T cells. Blast count starts to drop slightly after lymphodepleting chemotherapy and decreases rapidly after infusion of CAR T cells. CAR T cell count starts to rise exponentially shortly after infusion and reaches peak values at approximately day +10. After clearing tumor cells from patients’ blood, CAR T cell count drops again but remains detectable on a low level for several months. Depicted on the upper half are the kinetics of cytokine secretion and development of CRS/ICANS without intervention. Secretion of GM-CSF and IL-1β (light blue) rises almost immediately after infusion of CAR T cells and peaks rapidly followed by an equally rapid decrease, IL-6 secretion (purple) starts to increase shortly after and peaks at the same time as CRS (orange) is most likely to occur; after which, IL-6 drops but remains on an elevated level for some time. Peak level of IL-6 correlates to the severity of CRS. ICANS (grey) typically occurs a bit later than CRS and is connected to migration of CAR T cells to the neural compartment. Treatment of severe CRS consists of inhibition of IL-6 signaling with tocilizumab (indicated by black bars), a blocking monoclonal antibody targeting the membrane bound and soluble IL-6 receptor. After injection of tocilizumab, IL-6 decreases shortly but accumulates over time as elimination by receptor internalization is inhibited as well. Normalization of body temperature and amelioration of CRS symptoms can be observed immediately or within a few hours after administration of tocilizumab. In the case of severe CRS, the effect of anti-IL-6 therapy can wear off and multiple injections are required for treatment. Indication for subsequent application of tocilizumab is re-occurrence of fever which is followed by an increase in IL-6 and exacerbation of CRS symptoms. (Figure 5C) Pathophysiology of CRS/ICANS. Expansion of CAR T cells results in inflammation in the extracerebral compartment (orange), which activates resting macrophages into cytokine-producing macrophages (TNF, IL-6, etc.). These cytokines stimulate other macrophages to produce more cytokines in series, resulting in a vicious cycle of stimulation and cytokine secretion. This so-called macrophage activation syndrome (MAS) is the major trigger for CRS in CAR T cell therapy. During expansion, CAR T cells migrate through the blood–brain barrier (light brown) into the intracerebral compartment (grey) followed by activated macrophages evoking intracerebral MAS. Consecutively, these effector cells start to attack neurological tissue, leading to neurological damage, which is observed clinically as ICANS. CAR: chimeric antigen receptor. CAR T: chimeric antigen receptor T cells. CRS: cytokine release syndrome. ICANS: immune effector cell-associated neurotoxicity syndrome. TNF: tumor-necrosis factor. IL-1ß: Interleukin 1-beta. GM-CSF: granulocyte-macrophage-colony-stimulating factor.

### 11.8. B-Cell Aplasia

CD19-CAR-T cell-mediated B-cell aplasia results in reduced immunoglobulin levels, which requires treatment by IgG replacement. Due to the fundamental understanding of the immune system and the well-established technologies to purify antibodies from healthy donors, the immune protective function of the B-lineage compartment can be substituted by regular immunoglobulin infusions [149] to prevent infections and are associated with improved quality of life in antibody deficiency [203]. Hypogammaglobulinemia post CD19-CAR-T cell therapy seems to be more pronounced and cause more complications in children than in adults. Continued B-cell aplasia and subsequent hypogammaglobulinemia are linked to CD19-CAR performance and persistence, which is dependent on the product that was used for the treatment of the underlying disease [131,139]. Tisagenlecleucel incorporating a 4-1BB costimulatory domain tends to persist longer than axicabtagene ciloleucel with a CD28 costimulatory domain [2,3]. Only tisagenlecleucel is approved for the treatment in children and young adults (<25 years) [21]. Infectious complications post CD19-CAR-T cell therapy are of multifactorial origin. Strategic combinatorial medications including antiviral, antifungal and antibacterial therapy in a time- and risk-adapted approach can prevent infectious complications. IgG replacement on a regular basis can reduce the risk for high grade infections [131,204]. 

### 11.9. Relapse Patterns in Pediatric CD19-CAR-T Cell Therapy

The main cause of death after tisagenlecleucel treatment according to the ELIANA trial NCT02435849 is relapse at a rate of 68% (13/19 patients). Only one patient in the ELIANA trial (1/19 patients, 5%) died from a directly linked tisagenlecleucel toxicity that caused cerebral hemorrhage during coagulopathy in the context of CRS (15 days after infusion). The other patients died from infectious complications including HHV-6-caused encephalitis and systemic mycosis in association with prolonged neutropenia as well as from complications that occur subsequent to therapies of the primary disease [149]. Undoubtedly, many complications are rather caused by the poor clinical state of patients when they qualify for CD19-CAR-T cell therapy according to the current treatment criteria.

**Figure 6:**
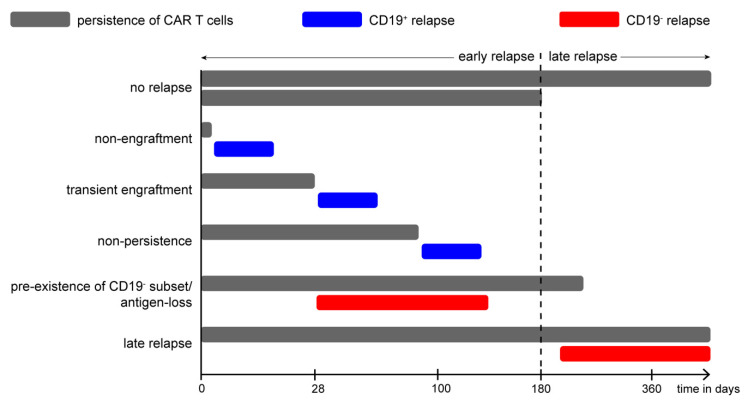
Relapse pattern after CD19-CAR-T cell therapy in BCP-ALL.

There are various strategies to reduce the probability of adverse events during CAR T cell therapy, but the most straight forward approach to improve safety is to implement CAR T cell therapy earlier in the treatment algorithm of r/r B-lineage ALL in children and young adults [172]. Consequently, patients will be in better condition than after intensive chemotherapy and allogeneic HSCT [2,176,177]. Reducing the risk of relapse would bring the highest impact on improving outcomes of CD19-CAR-T cell therapy, since most patients die from the leukemia recurrence, but not of CAR T cell induced complications. Therefore, addressing the question of “How to prevent relapse?” will bring most benefits to our patients, and understanding the current relapse pattern (illustrated in Figure 6) will direct the strategies that are most promising for improving patient outcome. 

The interplay of CD19^+^ B-lineage ALL blasts in childhood ALL and CD19-CAR-T cells define the relapse pattern after this highly potent targeted therapy. Early and late relapse may be distinguished at day +180 after CAR T cell infusion; however, the mechanism of relapse is strongly dependent **firstly** on the antileukemic performance [2] of the CAR T cells, and **secondly** on the pre-existence or development of CD19^−^ leukemic subsets as a result of the selection pressure [121]. In children with acute B-lineage ALL, the optimal scenario is the induction of complete molecular remission and maintenance beyond 6 months after CAR T cell infusion. These patients have a high chance of achieving long-term remission and leukemia-free survival. Patients whose CAR T cells have poor antileukemic performance of their CAR T cells, indicated in the figure by non-engraftment, transient engraftment, non-persistence and/or lack of exponential expansion, tend to develop CD19^+^ relapses. Without the selection pressure exerted by CD19-CAR-T cells, BCP-ALL blasts maintain CD19 expression. Patients whose CAR T cells have high antileukemic activity, as per the definition above, do not experience CD19^+^ relapse. Nonetheless, 10–20% of patients develop CD19^−^ relapses. There are two major independent mechanisms on how CD19^−^ relapse may occur. Pre-existence of CD19^−^ blasts prior to CD19-CAR-T cells at a very low frequency has been identified as a primary resistance mechanism [205], while the other mechanism is the development of CD19^−^ subsets over time via lineage switch or antigen escape (loss of the targeted epitope, alternative splicing of CD19 or downregulation of CD19) [30,84,206]. Grey: persistence of CAR T cells. Blue: CD19-positive relapse. Red: CD19-negative relapse. 

Relapse patterns post CD19-CAR-T cell therapy can be classified by the expression status of CD19, but remain elusive and difficult to predict. Nonetheless, there are known determinants of CD19^+^ and CD19^−^ relapses. The key discriminators are leukemic burden at the initiation of treatment with CD19-CAR-T cells (MRD > 10^−2^ versus MRD < 10^−3^), previous exposure to blinatumomab and duration of B-cell aplasia [207].

As known from alternative CD19-targeted therapies including CD19 antibody therapy and BiTE therapy using blinatumomab, insufficient leukemia control leads to an increased risk of CD19^−^ relapse and extramedullary leukemia formation, driven by the evolutionary pressure put on leukemia [16,82]. Incomplete clearance of BCP-ALL by blinatumomab may also predict resistance to CD19-CAR-T cell therapy [2,207]. In the beginning of CD19-CAR-T cell therapy, it was observed that patients with a higher leukemia burden would facilitate a better engraftment of CD19-CAR-T cells and thus CAR T cell therapy would be more successful. This early observation has been disproved in B-ALL. It has been demonstrated that patients with a lower leukemia burden show a favorable outcome [163]. 

Patients with a higher leukemia burden show a different relapse pattern to patients with a lower leukemia burden. Patients with a higher leukemia burden carry more leukemic blasts, leading to faster and more robust engraftment of CD19-CAR-T cells at the cost of more severe adverse events, such as CRS and ICANS [163]. Since the number of leukemic blasts is significantly higher, there is a greater chance of both the pre-existence and emergence of CD19 antigen immune escape variants. Thus, patients with a higher leukemic load tend to relapse with CD19^−^ leukemia. Conversely, patients with a lower leukemic burden show less rapid and robust engraftment of CD19-CAR-T cells and suffer from less severe adverse events. Due to the less robust or transient engraftment of CD19-CAR-T cells in these patients, CAR T cell performance may be reduced, and patients tend to experience CD19^+^ relapse (Figure 6) [207]. 

CD19-CAR-T cell performance and persistence can be monitored efficiently by standard flow cytometric evaluation of B-cell reconstitution. While continuous B-cell aplasia indicates persistence and functionality of CD19-CAR-T cells, patients with B-cell reconstitution at early time after CAR T cell treatment (prior to 6 months post infusion) tend to have a significantly increased risk for CD19^+^ leukemia recurrence [2,149,163]. However, some patients with co-existence of low B-cell counts and circulating CD19-CAR-T cells remain in remission, which may be partially attributed to the stronger resistance of physiologic B cells to CD19-CAR-T cells than leukemic blasts [2,149,163,207]. 

### 11.10. CAR T Cell Trials on Alternative Targets in B-Lineage Malignancies

Relapses can occur in over 60% of patients treated with CD19-CAR-T cells within the first 12 months, despite remarkable initial response rates [149,163]. The majority of these relapses are attributable to immune escape due to CD19 antigen loss or decreased expression [109,208,209]. This has lead to numerous investigations of alternative B-lineage markers including CD20 and CD22 as targets for CAR T cell therapy against B-cell lymphomas. Both CD20 and CD22 are highly expressed in B-cell lymphoblasts, with 50% and 80–90% expression, respectively [210]. CD20 has been extensively studied as a therapeutic target for the treatment of r/r B-cell non-Hodgkin’s lymphoma (NHL) and r/r B-ALL, with demonstrated preclinical and clinical efficacy [211]. The clinical success of CD20-CAR-T cells in adult B-cell lymphoma patients is moderate with high relapse rates (>80%), albeit showing favorable initial complete response rates (>50–70%) [212,213,214]. More promising is the preliminary data from a phase I CD22-CAR-T cell trial in children and young adults, with a 70% complete response rate and a median 6 month relapse-free survival, despite including multiply relapsed patients who had previously relapsed CD19_low_ or CD19^−^ after CD19-CAR-T cell therapy [84,181]. Unlike in CD19-CAR-T cells, relapses post CD22-CAR-T cell treatment occur mainly due to decreased CD22 expression (antigen downregulation) rather than antigen loss. However, this accentuates the common problem of immune escape as a mechanism of resistance to monovalent CAR T cell therapy and raises the question as to the long-term efficacy of CAR therapy beyond CD19. To counteract the risk of immune escape in B-lineage cancers, bivalent CAR T cells simultaneously targeting CD19-CD22, and CD19-CD20 have been developed and tested in phase I trials [80,111,215,216,217]. Antileukemic activities and complete remission rates were comparable to monovalent CAR T cells; however, long-term efficacy was not attained due to relapse. Interestingly, the relapses were due to loss or decreased expression of CD19, and not CD22, indicative of a biased selective pressure on CD19 [80]. In another trial utilizing CD19-CD20 tandem-CAR-T cells, CD19 expression was retained in all relapsed patients [217]. These results highlight the presence of multiple resistance mechanisms to CAR T cell therapy. 

Driven by the need for additional target antigens to reduce the risk of antigen escape, CD37 and CD79B have emerged as promising novel CAR T cell targets for B-cell malignancies. Both CD37 and CD79B are highly expressed across multiple types of B-cell malignancies. Both CD37- and CD79B-CAR T cells have shown specific and effective antitumor activities in vitro as well as in vivo, which supports further clinical development [87,218,219]. At the time of this review, there is one phase I trial for both anti-CD37- and CD79B-CAR-T cell products in the early phase of recruitment (Table 2).

### 11.11. CAR T Cell Therapy for T Cell Malignancies

Among of the most challenging cancers to treat with CAR T cells are T-lineage-derived malignancies. The main reason for this challenge is the co-expression of the target antigens on physiological T cells and progenitors thereof. Targeting of T-lineage-associated antigens leads to fratricide of CAR T cells and physiological T cells, the key immune cell subset of the adaptive immune compartment. Further, separating physiological T cells from malignant T cells during CAR T cell manufacturing has not been solved satisfactorily. There are no T cell-exclusive target antigens that can be targeted with CAR T cells without severely compromising the T cell compartment. However, targeting of CD2, CD5, CD7 and CD38 as well as the constant chains of the T cell receptor TRCB1 or TRCB2 have been used successfully in preclinical models [89,90]. Impressive CD7-CAR-T cell responses (90% remission induction rate) in patients with acute T cell leukemias were reported from the Chinese trial NCT04689659, with 15 out of 20 patients being in remission after a median follow-up of 6 months. CAR T cell persistence was confirmed in month 6 after CAR T cell infusion of 0.5–1 × 10^6^/kgBW. Interestingly, physiological CD7-negative T cells expanded and compensated for treatment-related T cell immunodeficiency [91]. Larger cohorts need to be treated to understand the current value of CAR T cell therapy in T-lineage malignancies. 

### 11.12. CAR T Cell Therapy for AML

The lack of cancer-specific antigens is the fundamental biological obstacle limiting the application of CAR T cell therapy in AML. Although AML blasts express various cell surface antigens such as CD33, CD123, CD38 and CLL-1, against which CAR T cells have been developed, these antigens are also expressed by hematopoietic stem or progenitor cells (HSPCs) [92]. Therefore, on-target off-tumor toxicity on HSPCs of these CAR T cells is of great concern, although they have shown potent antitumor activity in preclinical models [93,94]. Prolonged myeloablation resulting from on-target off-tumor toxicity on HSPCs can induce fatal infections in neutropenic fever and bleeding disorders. Strategies to facilitate CAR T cell therapy in AML include using CAR T cells as a remission induction therapy and rescuing the hematopoiesis by allogeneic HSCT. Further, myelotoxicity by CAR T cells can be terminated by CAR T cell ablation via suicide switches, and can be circumvented by generation of gene knockout of the targeted antigen (e.g., CD33) in rescue hematopoietic stem cell grafts [116]. 

Currently, CAR T cell products for AML that are in clinical trials mostly target CD33, CD38, CD123 and CLL-1 (Table 2). There are limited clinical data published at this stage to allow a thorough appreciation of the safety and efficacy profile of these CAR T cell products, although promising clinical responses have been reported, with myeloablation managed by HSCT [116]. 

### 11.13. CAR T Cell Therapy in Solid Tumors

In comparison to hematological cancers, solid tumors pose several unique challenges to CAR T cell therapy. Solid tumors encompassing the majority of cancers exhibit high levels of intrinsic tumor heterogeneity. CAR T cells that target only one antigen therefore are unable to recognize all the cancer cells in the tumor. Target antigens under investigation in solid tumors are always co-expressed at lower levels in vitally essential tissues. Consequently, it is inevitable to cause on-target off-tumor toxicities on healthy tissues [95]. Another major challenge is the highly immunosuppressive tumor microenvironment (TME) inducing T cell inactivation and dysfunction. Thus far, CAR T cell therapies in solid tumors lack clinical efficacy and have caused severe toxicities [31]. 

Pediatric brain tumors remain the leading cause of cancer-related death in children. CAR T cells have been developed to target the antigens B7-H3 (CD276), GD2, EGFR, IL13Ra2 and HER2 in a range of brain cancers such as medulloblastoma, glioma and ependymoma [220]. GD2-CAR-T cells have shown promising antitumor activity in neuroblastomas and sarcomas [221]. B7-H3 has been characterized as a pan-cancer antigen overexpressed in a variety of solid tumors including neuroblastoma and pediatric sarcomas, for which CD276-CAR-T cells are being investigated [222]. Supported by encouraging preclinical results, these CAR T cells have progressed to phase I clinical trials to assess their safety (Table 3). 

## 12. Novel CAR T Technologies—The Antigen Question

The remarkable clinical success of CD19- and BCMA-CAR-T cell therapy [2,3,223] is the result of three decades of continuous research effort [35]. Today, the focus has shifted to removing the roadblocks in CAR T cell therapy to facilitate its application in acute myeloid leukemia (AML) and solid cancers. The main goals are to increase the clinical efficacy of CAR T cells while improving safety profiles and reducing the treatment costs [172]. 

Current limitations in CAR T cell therapy are mainly defined by how conventional CAR T cells operate. Most of the key obstacles are defined by the targeted antigens. The ideal target antigens are homogeneously expressed on all cancer cells, at sufficient levels above the CAR T activation threshold, and are significantly overexpressed in cancerous tissue with low expression in healthy tissues, and no expression in vitally essential tissues to spare toxicities [132]. The pursuit of the perfect antigen is rather far-fetched. The main effort to improve CAR T cell therapy in B-lineage malignancies is to efficiently target beyond CD19 [111,150,181]. 

As discussed above, the major cause of treatment failure and subsequent death of patients who received CAR T cell therapy in B-lineage cancers is relapse. To address antigen-negative relapse, which occurs in approximately 20% of CD19-CAR-T cell-treated patients (in BCP-ALL) [2], multitargeted CAR T cell products have been developed and are used in various formats as tandem CAR constructs or bicistronic or even tri-valent CAR constructs [80,84,108]. Clinical success of combinatorial CAR constructs is to be demonstrated and first data indicate that the clinical benefit is far less than anticipated. Dual CAR constructs did not show the same efficacy as two single-CAR T cell constructs for the same targeted antigens. The reduced potency seems to increase the chance of antigen low-positive relapse and immune escape variants which may not develop with more potent monotargeted CAR T cell therapies [181]. Further, sequential CD22 targeting in patients who experienced CD19-negative relapse after CD19-CAR-T cell therapy was associated with the emergence of CD22_low_ relapse [84] and did not solve the antigen problem either. 

Altogether, the fundamental challenge in CAR T cell therapy is to generate highly potent and safe CAR T cell products targeting non-B-lineage-derived cancers in a clinical setting. There are numerous preclinical studies that have demonstrated efficacy in various cancer models including AML [224], melanoma, lung cancer, brain cancer, osteosarcoma, Ewing sarcoma, prostate cancer [225], pancreatic cancer [226], and liver cancer [227]; however, clinical translation has not been successful [222]. Major advancements in CAR T cell therapy are expected, once multitargeted approaches facilitate the treatment of antigen heterogenous cancers.

### 12.1. Indirect CAR Technologies

**Figure 7:**
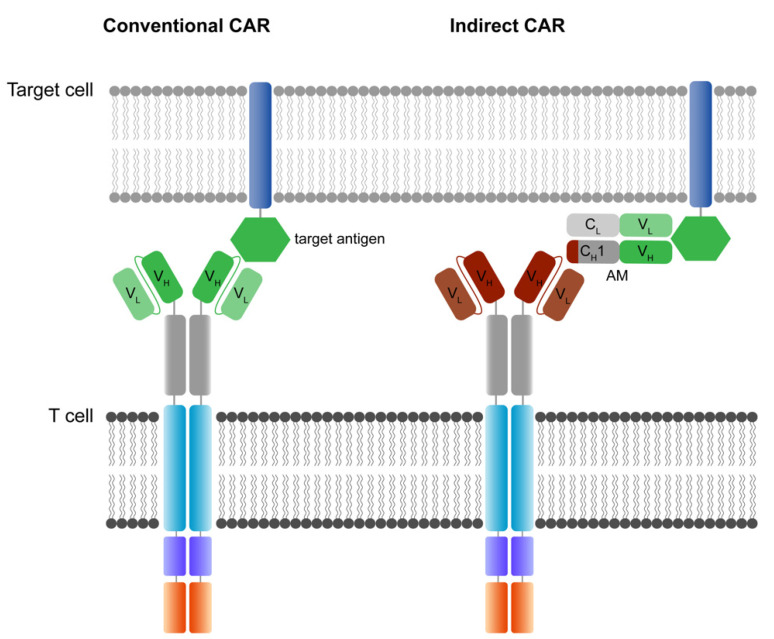
The designs of conventional and indirect CAR T cell technologies.

One elegant way to overcome immune escape in CAR T cell therapy is a multitargeted approach utilizing indirect CAR technologies. Adapter CAR technologies are two-component CAR technologies. The first component is comprised of a universal CAR T cell and the second component consists of adapter molecules that link the CAR receptors to the overexpressed tumor-associated antigen, leading to the recruitment and engagement of the CAR-expressing cells (Figure 7). The effector functions are the same as in conventional CAR T cells.

Conventional CAR T cells directly recognize the targeted antigen with their antigen recognition domain, which in most CAR constructs is a scFv. Indirect CAR technologies aim to highly specifically target exclusive *neo-antigens* incorporated on the adapter molecules as tags, such as peptides or LLE or chemical agents, on the adapter molecule to facilitate a clean off-state during the absence of the activating adapter molecules and a clean on-state during the presence of the adapter molecules and the target antigens. In the case of using non-exclusive antigens, such as alpha-fetoprotein cross reactivity and blocking phenomena can be induced. For the adapter CAR T cell system to function properly, the adapter CAR effector cell, the adapter molecule, and the target need to assemble correctly. This mechanism is more complex and dependent on more variables compared to the direct targeting of a conventional CAR. On the other hand, adapter CAR technologies are versatile and can facilitate features which are not achievable with conventional CAR targeting. These features include the safety and efficacy aspects determined by the nature of the on and off switches, the universal targeting (one CAR for all antigens), combinatorial targeting and more physiologic recruitment of the CAR-expressing cells. To date, conventional and indirect CAR technologies shall be regarded as complementary to each other. If adapter CAR technologies reach the same level of efficacy as conventional CAR technologies, they are likely to substitute conventional CARs for the clear advantages arising from the flexible technology.

There are numerous sophisticated adapter CAR technologies that have been developed over the last decade with distinct functional properties. They may be grouped into different CAR systems: (**I**) CARs that make use of the high-affinity CD16 (FCGR3A) V158 variant [228] in combination with antibodies or Fc-engineered antibodies [229], (**II**) scFv-based CARs targeting a tag on the adapter molecules, such as a the chemical compound FITC [230,231], peptides [232,233], alpha-fetoprotein (AFP) [234] or naturally occurring vitamins such as biotin [132,235], and (**III**) non-scFv-based binding domains such as streptavidin-derived avidin [236] or leucine zippers [237], and (**IV**) ligand-based CARs that are recruited via bivalent adapter molecules [238]. Putting aside the details in the distinct mode of actions, adapter CAR technologies may be considered to be advanced versions of antibody-dependent cellular cytotoxicity. 

All listed strategies aim to overcome the obstacles of conventional CAR T cell therapy and can facilitate universal targeting (one CAR for all target antigens), combinatorial targeting and transient targeting. The switch-on and switch-off mechanism provides both enhanced safety and efficacy.

In the CAR T cell community, the main criticism arises from the added physiological complexity of adapter CAR systems. Conventional CAR T cells are always equipped with the recognition domain, whereas adapter CAR T cells are “*blind*” without the adapter molecules. Thus, adapter CAR T cells need to assemble correctly with the adapter molecule and the targeted antigen before they become functional. There is clear evidence that the adapter molecule format and size will impact on the distribution and elimination kinetics, the pharmacokinetics and pharmacodynamics [239]. Human IgG antibodies are mainly eliminated via intracellular catabolism by lysosomal degradation after pinocytosis, unspecific endocytosis, or by receptor-mediated endocytosis [240], whereas low-molecular-weight antibody fragments or fusion proteins below 70 kDa [241] are filtered and usually reabsorbed and metabolized in the proximal tubule of the nephron [239]. 

Certain body compartments are less accessible than others and it remains uncertain how adapter molecules may penetrate into the CNS [242], the testicles, and cancer tissues [243,244,245]. In many cancers, such as leukemias, primary brain cancers and brain metastasis, intracranial anticancer activity will be crucial for long-term tumor control [2]. On the other hand, antibodies have been shown to be functional beyond the blood–brain barrier such as immune checkpoint inhibitors (antibodies) and blinatumomab; however, their ability to penetrate the CNS is discussed controversially [245,246,247]. 

The advantages of adapter CAR technologies though are apparent. They provide solutions to overcome the major limitations in CAR T cell therapy. With regard to tissue penetration of adapter molecules, the reduced size of adapter molecules in the Fab or scFv formats have been used successfully in various adapter CAR technologies [232,248]. There are various strategies to overcome the blood–brain barrier in order to reach effective concentrations in cancer tissues, the testicular tissue and the CNS. Firstly, there is an obvious pharmacodynamic advantage of adapter molecules (for adapter CAR T cells) over blinatumomab. Adapter molecules can be administered at significantly higher doses than BiTEs, because no relevant unspecific activation of adapter CAR T cells is induced by adapter molecules in absence of the target [132,232]. In contrast, the maximum tolerated dose of blinatumomab defined by the study “Clinical Study With Blinatumomab in Patients With Relapsed/Refractory Diffuse Large B-cell Lymphoma (DLBCL)” (NCT01741792) in adults is 60 ug/m^2^/day, reaching serum concentrations of 3 ng/mL, up to a maximum applied dose of 90 ug/m^2^/day, reaching 3.5 ng/mL [249]. The recommended blinatumomab dose for the treatment of BCP-ALL in children is 5–15 ug/m^2^/day, reaching serum concentrations of 0.6 ng/mL [167]. By increasing the dose significantly for instance by 1000-fold to more than 100 ng/mL, the adapter molecule penetration into the CNS and cancer tissues would be increased [243]. Secondly, intrathecal, intraventricular or intratumoral applications [250] of adapter molecules and CAR T cells have been shown for mesothelioma [251] and glioblastoma [252] and appear to be feasible and would help to overcome the blood–brain barrier or blood-cancer barrier through changing the application route [190,253]. Intrathecal application of chemotherapy in the treatment of leukemias is a standard procedure for both treatment of leukemic CNS involvement as well as CNS prophylaxis [254]. 

The low clinical tolerability of BiTEs clearly limits the potency of these fantastic new drugs, and if higher concentrations could be tolerated, the advantages of complex adapter CAR technologies compared to bispecific immune cell recruiting fusion proteins would be significantly reduced. In this scenario, BiTEs would most likely make the race for many applications as they are off-the-shelf products, need fewer complex infrastructures, making them cheaper to produce, handle and apply, and still have shown great clinical efficacy, despite being underdosed for toxicity reasons. The real-life clinical experience, however, will not support using BiTEs at their optimal concentrations in human patients [249]. Despite that adapter CAR T cell technologies will have to prove their superiority over blinatumomab in resistant CD19^+^ leukemias and lymphomas, they have a very promising prospect as adapter CAR technologies have the chance to overcome the unspecific BiTE toxicities beyond CD19 targeting. Bispecific antibodies including BiTEs have not been convincingly successful in other leukemias such as AML [255], even though they demonstrated promising in vitro efficacy in primary AMLs at 5 ng/mL [256].

### 12.2. Technologies to Improve the Safety of CAR T Cells

Major CAR T cell infusion-related side effects comprise systemic inflammatory responses derived from rapid T cell expansion and on-target off-tumor effects. By expressing a targetable truncated antigen on CAR T cells (e.g., EGFR) and infusion of the corresponding antibodies (e.g., cetuximab), the elimination of the engineered CAR T cells can be achieved [257]. Another safety switch makes use of the inducible Caspase 9 (iCasp9) suicide gene, which offers a fast onset and more specific elimination of highly activated CAR T cells with high levels of transgene expression [258,259]. Sterner et al. studied granulocyte-macrophage colony-stimulating factor (GM-CSF) disruption by CRISPR/Cas9 in preclinical murine models and showed that neutralizing GM-CSF by lenzilumab is a potential strategy to abrogate CD19-CAR-T-related CRS without inhibition of CAR T cell function [260]. 

Discriminating normal tissues from cancer cells by the usage of logic gating of CARs can eliminate the on-target off-tumor effects. One example is the synNotch gating [261]. A synNotch receptor recognizes the first antigen which triggers the expression of a CAR toward a second tumor antigen. The recognition of both antigens enables the activation of T cells. The slow activation and degradation kinetics are the major limitations for further clinical application. Another strategy for combined sensing of two or more tumor antigens is to split the primary CD3ζ and the costimulatory domain into two separate chimeric receptors that are introduced into the same T cell [258,262].

Recent studies have substantiated the feasibility of controlling CAR functions via small-molecule interactions. Modulation of CAR functions can be achieved by using dimerizing agents as on switches and off switches [263], as well as on switches leading to a conformational change in the variable recognition domains induced by methotrexate that functions as an off switch [264]. In preclinical models, CAR T cell functions were shown to be tightly controlled via a pharmacological on/off switch using the tyrosine kinase inhibitor dasatinib. Dasatinib can abrogate CAR-mediated effector functions such as cytokine secretion, proliferation, and cytolysis rapidly with regular dosing. After termination of dasatinib exposure, CAR functions were fully restored, and even synergistic effects potentiating CAR function have been observed. In contrast to corticosteroid therapy in CRS, application of dasatinib would be distinguished from the other strategies to ablate CAR T cells [265,266].

With respect to the current developments in CAR T cell therapy, translational and practical approaches will help to improve patient safety. Optimizing the CD4/CD8 composition in CAR T cell products and reducing the number of accessory cells can reduce life-threatening CRS and ICANS while retaining the potency of CAR T cell products [181,267]. The development of ICANS in patients treated with the CD19-CAR-T cell product axicabtagene ciloleucel is associated with the number of accessory cells in the product and not with the number of CAR T cells. These cells may be referred to as ICANS-associated cells and carry a distinct monocyte-like transcriptional signature [267]. 

### 12.3. Armored Modules to Increase CAR T Cell Performance in TME

The production of inhibitory cytokines is employed by the tumor cells to create an immunosuppressive tumor microenvironment (TME) and evade the anticancer immune response. To protect CAR T cells from the immunosuppressive TME, engineered cytokine factor blockades have been developed in the format of cytokine switch receptors or dominant-negative receptors (DNR). For example, interleukin-4 (IL-4) is a key cytokine that controls the differentiation of T cells to the TH2 effector cells, which promotes and maintains an immunosuppressive environment and abrogates the anticancer immune response [268]. IL-4-IL-7 switch receptors transform the inhibitory IL-4 signal into a proinflammatory IL-7 signal with proliferative potential, promoting fitness, youth, and survival of T cells [269]. Another important immunosuppressive cytokine is TGFβ, which has been associated with in tumor progression and metastasis formation in several types of cancers. Upon expression of the DNR TGF-βRII in CAR T cells, enhanced cytokine secretion, resistance to exhaustion and improved long-term in vivo persistence have been observed in human prostate cancer mouse models of PSMA-CAR-TGF-ßRII-DNR-T cells [270]. 

Immune checkpoint inhibition by therapeutic antibodies unleashes the T cell antitumor immunity of T cells. Multiple strategies to achieve engineered PD-1 checkpoint blockade have been developed. Engineered PD1-CD28 switch receptors [271], secretion of blocking PD-1-targeted scFv [272] or antibodies by CAR T cells [273], shRNA knockdown of PD-1 and DNR of PD-1 have been shown to overcome immune checkpoint inhibition [274]. These potentiating CAR technologies and strategies have demonstrated the ability to increase the CAR effector functions in preclinical models [275,276]. Multiple genetic receptor modifications in CAR T cells have already reached the clinic, as exemplified by the “Study of CRISPR-Cas9 Mediated PD-1 and TCR Gene-knocked Out Mesothelin-directed CAR-T Cells in Patients With Mesothelin Positive Multiple Solid Tumors” (NCT03545815) that demonstrates feasibility with proof-of-concept experiments in preclinical models and with early preliminary clinical response data in a limited number of patients demonstrating feasibility [276]. Furthermore, advanced gene editing technology fuels the endeavors of donor-independent CAR T cell therapy by enabling gene knockouts of the TRAC and ß-microglobulin gene, which consequently eliminate the T cell receptors and the MHCs, leading to abrogation of GvHD and prevention of T cell-mediated rejections, respectively [277,278]. These genetic modifications may promote donor-independent allogeneic application of CAR therapeutics in the future. However, the T cell receptors and immune checkpoint receptors are important homeostatic receptors involved in the physiological functions of T cells, for which further investigation to fully understand the impact of these modifications on co-signaling pathways is imperative in order to ensure patient safety, and to create the most efficacious transgenic T cell therapy [279,280]. Site-specific integration of CAR transgenes into the TRAC locus using engineered endonucleases or CRISPR/Cas9 in combination with AAV templates or single-stranded DNA and electroporation for gene delivery has been established [63,281]. Integration of the CAR in the TRAC locus under the expression modulation of the TcR has demonstrated an activation-dependent transgene expression, which has shown to be advantageous compared to constitutive CAR expression in preclinical models and a step forward towards universal allogeneic CAR T cell therapy by disruption of the endogenous TcR [63,282]. However, to date, there is no proof that this new approach will improve CAR T cell therapy in humans. Despite the amazingly sophisticated technology, CARs will not be able to substitute the constitutive signaling function of the TcR [280]. Other strategies attempt to integrate the CARs in various ways to achieve the assembly within the native CD3 T cell receptor complex and make use of the TcR machinery [283,284], but these approaches are accompanied by other obstacles, such as TcR mispairing, overstimulation, and lack of space for antigen-specific receptor optimization.

CAR T cells engineered to secrete cytokines such as IL-12 [285] and IL-18 [286] or express tethered IL15-IL15RA [287] have proven to augment CAR T cell functions and are regarded as the fourth generation of CAR T cells. CARs that induce cytokine secretion upon recognition of the antigens are referred to as TRUCKs [62] and may overcome the toxic limitations of highly and constitutively expressed potent cytokines.

## 13. Conclusions

CAR T cell therapy has become a highly valued treatment in pediatric patients with r/r B-lineage malignancies. Thus far, CD19 is the best target antigen for CAR T cell therapy which has lead to cures for patients who were considered incurable. This unprecedented clinical success has ignited worldwide efforts to broaden the application of CAR T cell therapy beyond targeting CD19. On the other hand, we have learned our lessons from CD19-CAR-T cell therapy [288] and recognized the drawback that every new CAR, targeting a different antigen other than CD19, will need to go through a rigorous and lengthy development and optimization program. Identifying the best suitable target antigens for each cancer and solving the antigen challenges for CAR T cell therapy are the most important strategies in the development of novel CAR T cell therapies [132,289]. 

Furthermore, economic aspects constantly change the competitive landscape of adoptive cell therapy. Despite the remarkable complete response rates in CD19-CAR-T cell therapy in children and young adults [2], it has been argued that the initial treatment costs and secondary costs are too high, and strategies to reduce manufacturing costs [290], treatment costs and secondary costs such as immunoglobulin replacements have to be carefully addressed [291,292]. We need to succeed in increasing the potency and safety of CAR T cell products and expand CAR T cell-based immunotherapy to other cancers. Next-generation CAR T cell technologies, including adapter CAR technologies, have the chance to overcome some of the current clinical and economic limitations and transform CAR T cell therapy into a treatment platform with versatile functions and applications in cancer and beyond.

## Figures and Tables

**Table 1:** FDA-approved CAR T cell products.

Name	Target Antigen	Brand	FDA Approval	Indications
Tisagenlecleucel	CD19	Kymriah	August 2017 May 2018	r/r B-cell precursor ALL, r/r large B-cell lymphoma
Axicabtagene ciloleucel	CD19	Yescarta	October 2017 March 2021	r/r large B-cell lymphoma r/r follicular lymphoma
Brexucabtagene autoleucel	CD19	Tecartus	July 2020 October 2021	r/r MCL (July 2020) r/r B-cell precursor ALL (Oct 2021)
Lisocabtagene maraleucel	CD19	Breyanzi	February 2021	r/r large B-cell lymphoma
Idecabtagene vicleucel	BCMA	Abecma	March 2021	r/r MM
Ciltacabtagene autoleucel	BCMA	Carvykti	February 2022	r/r MM

**Table 2:** CAR T cell trials for B-lymphoid leukemias (non-CD19 targeting) and AML.

CAR Target	Condition Treated	Eligible Age	Status	ClinicalTrials.gov ID
CD20	B-cell Non-Hodgkin’s lymphomas	≥18	Recruiting	NCT03277729
CD20	B-cell lymphoma r/r to anti-CD19-CAR-Ttherapy	14 to 70	Unknown	NCT04036019
CD20	Lymphomas r/r to chemotherapy	≥18, <90	Unknown	NCT01735604
CD22	r/r B-cell lymphoma/leukemia	3 to 39	Recruiting	NCT02315612
CD22	B-ALL	1–30	Recruiting	NCT04088864
CD22	B-ALL	1–24	Recruiting	NCT02650414
CD22	r/r B-ALL	15–70	Recruiting	NCT04150497
CD19, CD20	r/r B-cell lymphoma/leukemia	16–70	Completed	NCT03097770
CD19, CD20	r/r B-cell lymphoma/leukemia	18–70	Active, not recruiting	NCT03019055
CD19, CD20	r/r B-cell lymphoma/leukemia	18–70	Recruiting	NCT04007029
CD19, CD20	r/r B-ALL	1–39	Recruiting	NCT04049383
CD19, CD22	r/r B-cell lymphoma/leukemia	≥18	Recruiting	NCT03233854
CD19, CD22	r/r B-cell lymphoma/leukemia	3–39	Recruiting	NCT03448393
CD19, CD22	r/r B-cell lymphoma/leukemia	6 months to 70	Recruiting	NCT04029038
CD19, CD22	B-cell lymphoma/leukemia	≤30	Recruiting	NCT03330691
CD19, CD22	r/r B-ALL	1–30	Recruiting	NCT03241940
CD37	B and T cell lymphoma/leukemia	≥18	Recruiting	NCT04136275
CD79B	r/r B-ALL, B-cell NHL	No age limit	Not yet recruiting	NCT04609241
CD33	AML	1–35	Recruiting	NCT03971799
CD123	AML	≥12	Recruiting	NCT02159495
CD123	AML	18–65	Recruiting	NCT03190278
CD123	AML	18–70	Recruiting	NCT04014881
CD123	AML	≥18	Active, not recruiting	NCT03766126
CD33, CLL-1, CD123	AML	6 months to 75	Recruiting	NCT04010877
CLL-1	AML	≤75	Recruiting	NCT04219163
CD38	AML	6–65	Recruiting	NCT04351022
CD33, CLL-1	AML/MDS/MPN/CML	No age limit	Recruiting	NCT03795779

**Table 3:** CAR T cell trials for pediatric solid tumors.

CAR Target	Condition Treated	Eligible Age	Status	ClinicalTrials.gov ID
B7-H3	Pediatric CNS tumors	1–26	Recruiting	NCT04185038
B7-H3	Pediatric solid tumors	≤26	Recruiting	NCT04483778
B7-H3	Solid tumors	1–75	Recruiting	NCT04432649
GD2	DIPG/high grade glioma	12 months to 18	Recruiting	NCT04099797
GD2	DIPG/DMG	2–30	Recruiting	NCT04196413
GD2	Osteosarcoma, neuroblastoma	≤35	Recruiting	NCT04539366
GD2	Neuroblastoma	12 months to 25	Recruiting	NCT03373097
GD2	Neuroblastoma, sarcoma	1–74	Recruiting	NCT03635632
GD2	Osteosarcoma, neuroblastoma	18 months to 18	Recruiting	NCT03721068
EGFR	Pediatric CNS tumors	≥15 and ≤26	Recruiting	NCT03638167
EGFR	Pediatric solid tumors	1–30	Recruiting	NCT03618381
EGFRvIII	Hematological and solid tumors	4–70	Recruiting	NCT03638206
HER2	Pediatric CNS tumors	1–26	Recruiting	NCT03500991
HER2	CNS tumors	≥3	Recruiting	NCT02442297
IL13Ra2	Pediatric CNS tumors	4–35	Recruiting	NCT04510051
IL13Ra2	Glioma	12–75	Recruiting	NCT02208362

## Data Availability

Not applicable.

## References

[1] Singh A.K., McGuirk J.P. (2020). CAR T cells: Continuation in a revolution of immunotherapy. Lancet Oncol..

[2] Maude S.L., Frey N., Shaw P.A., Aplenc R., Barrett D.M., Bunin N.J., Chew A., Gonzalez V.E., Zheng Z., Lacey S.F. (2014). Chimeric Antigen Receptor T Cells for Sustained Remissions in Leukemia. N. Engl. J. Med..

[3] Neelapu S.S., Locke F.L., Bartlett N.L., Lekakis L.J., Miklos D.B., Jacobson C.A., Braunschweig I., Oluwole O.O., Siddiqi T., Lin Y. (2017). Axicabtagene Ciloleucel CAR T-Cell Therapy in Refractory Large B-Cell Lymphoma. N. Engl. J. Med..

[4] Spadoni C. (2018). Pediatric Drug Development: Challenges and Opportunities. Curr. Res. Clin. Exp..

[5] Joseph P.D., Craig J.C., Caldwell P.H.Y. (2015). Clinical trials in children. Br. J. Clin. Pharmacol..

[6] Kakaje A., Alhalabi M., Ghareeb A., Karam B., Mansour B., Zahra B., Hamdan O. (2020). Rates and trends of childhood acute lymphoblastic leukaemia: An epidemiology study. Sci. Rep..

[7] Siegel R.L., Miller K.D., Fuchs H.E., Jemal A. (2021). Cancer Statistics, 2021. CA Cancer J. Clin..

[8] Terwilliger T., Abdul-Hay M. (2017). Acute lymphoblastic leukemia: A comprehensive review and 2017 update. Blood Cancer J..

[9] Inaba H., Mullighan C.G. (2020). Pediatric acute lymphoblastic leukemia. Haematologica.

[10] Reismüller B., Peters C., Dworzak M.N., Pötschger U., Urban C., Meister B., Schmitt K., Dieckmann K., Gadner H., Attarbaschi A. (2013). Outcome of children and adolescents with a second or third relapse of acute lymphoblastic leukemia (ALL): A population-based analysis of the Austrian ALL-BFM (Berlin-Frankfurt-Münster) study group. J. Pediatr. Hematol. Oncol..

[11] Lemal R., Tournilhac O. (2019). State-of-the-art for CAR T-cell therapy for chronic lymphocytic leukemia in 2019. J. ImmunoTher. Cancer.

[12] Schuster S.J., Bishop M.R., Tam C.S., Waller E.K., Borchmann P., McGuirk J.P., Jäger U., Jaglowski S., Andreadis C., Westin J.R. (2018). Tisagenlecleucel in Adult Relapsed or Refractory Diffuse Large B-Cell Lymphoma. N. Engl. J. Med..

[13] Pui C.-H., Evans W.E. (2013). A 50-year journey to cure childhood acute lymphoblastic leukemia. Semin. Hematol..

[14] Brissot E., Rialland F., Cahu X., Strullu M., Corradini N., Thomas C., Blin N., Thebaud E., Chevallier P., Moreau P. (2016). Improvement of overall survival after allogeneic hematopoietic stem cell transplantation for children and adolescents: A three-decade experience of a single institution. Bone Marrow Transplant..

[15] Handgretinger R., Zugmaier G., Henze G., Kreyenberg H., Lang P., Von Stackelberg A. (2011). Complete remission after blinatumomab-induced donor T-cell activation in three pediatric patients with post-transplant relapsed acute lymphoblastic leukemia. Leukemia.

[16] Patrick S., Peter L., Gerhard Z., Martin E., Hermann K., Kai-Erik W., Judith F., Matthias P., Heiko-Manuel T., Christina K. (2014). Pediatric posttransplant relapsed/refractory B-precursor acute lymphoblastic leukemia shows durable remission by therapy with the T-cell engaging bispecific antibody blinatumomab. Haematologica.

[17] Lee D.W., Kochenderfer J.N., Stetler-Stevenson M., Cui Y.K., Delbrook C., Feldman S.A., Fry T.J., Orentas R., Sabatino M., Shah N.N. (2015). T cells expressing CD19 chimeric antigen receptors for acute lymphoblastic leukaemia in children and young adults: A phase 1 dose-escalation trial. Lancet.

[18] Kebriaei P., Singh H., Huls M.H., Figliola M.J., Bassett R., Olivares S., Jena B., Dawson M.J., Kumaresan P.R., Su S. (2016). Phase I trials using Sleeping Beauty to generate CD19-specific CAR T cells. J. Clin. Investig..

[19] Turtle C.J., Hanafi L.-A., Berger C., Gooley T.A., Cherian S., Hudecek M., Sommermeyer D., Melville K., Pender B., Budiarto T.M. (2016). CD19 CAR–T cells of defined CD4+:CD8+ composition in adult B cell ALL patients. J. Clin. Investig..

[20] Turtle C.J., Hay K., Hanafi L.-A., Li D., Cherian S., Chen X., Wood B., Lozanski A., Byrd J.C., Heimfeld S. (2017). Durable Molecular Remissions in Chronic Lymphocytic Leukemia Treated With CD19-Specific Chimeric Antigen Receptor–Modified T Cells After Failure of Ibrutinib. J. Clin. Oncol..

[21] (2017). KYMRIAH^®^ (Tisagenlecleucel) [Package Insert].

[22] (2017). YESCARTA^®^ (Axicabtagene Ciloleucel) [Package Insert].

[23] (2021). BREYANZI^®^ (Lisocabtagene Maraleucel) [Package Insert].

[24] (2021). TECARTUS^®^ (Brexucabtagene Autoleucel) [Package Insert].

[25] (2021). ABECMA^®^ (Idecabtagene Vicleucel) [Package Insert].

[26] Quintás-Cardama A. (2019). What CAR Will Win the CD19 Race?. Mol. Cancer Ther..

[27] Schuster S.J., Maziarz R.T., Rusch E.S., Li J., Signorovitch J.E., Romanov V.V., Locke F.L., Maloney D.G. (2020). Grading and management of cytokine release syndrome in patients treated with tisagenlecleucel in the JULIET trial. Blood Adv..

[28] Sheth V.S., Gauthier J. (2021). Taming the beast: CRS and ICANS after CAR T-cell therapy for ALL. Bone Marrow Transplant..

[29] Wudhikarn K., Palomba M.L., Pennisi M., Garcia-Recio M., Flynn J.R., Devlin S.M., Afuye A., Silverberg M.L., Maloy M.A., Shah G.L. (2020). Infection during the first year in patients treated with CD19 CAR T cells for diffuse large B cell lymphoma. Blood Cancer J..

[30] Majzner R.G., Mackall C.L. (2018). Tumor Antigen Escape from CAR T-cell Therapy. Cancer Discov..

[31] Wagner J., Wickman E., DeRenzo C., Gottschalk S. (2020). CAR T Cell Therapy for Solid Tumors: Bright Future or Dark Reality?. Mol. Ther..

[32] Chen G.M., Chen C., Das R.K., Gao P., Chen C.-H., Bandyopadhyay S., Ding Y.-Y., Uzun Y., Yu W., Zhu Q. (2021). Integrative Bulk and Single-Cell Profiling of Premanufacture T-cell Populations Reveals Factors Mediating Long-Term Persistence of CAR T-cell Therapy. Cancer Discov..

[33] Kasakovski D., Xu L., Li Y. (2018). T cell senescence and CAR-T cell exhaustion in hematological malignancies. J. Hematol. Oncol..

[34] Martinez M., Moon E.K. (2019). CAR T Cells for Solid Tumors: New Strategies for Finding, Infiltrating, and Surviving in the Tumor Microenvironment. Front. Immunol..

[35] Gross G., Waks T., Eshhar Z. (1989). Expression of immunoglobulin-T-cell receptor chimeric molecules as functional receptors with antibody-type specificity. Proc. Natl. Acad. Sci. USA.

[36] Milone M.C., Fish J.D., Carpenito C., Carroll R.G., Binder G.K., Teachey D., Samanta M., Lakhal M., Gloss B., Danet-Desnoyers G. (2009). Chimeric Receptors Containing CD137 Signal Transduction Domains Mediate Enhanced Survival of T Cells and Increased Antileukemic Efficacy In Vivo. Mol. Ther..

[37] Gong M.C., Latouche J.-B., Krause A., Heston W.D., Bander N.H., Sadelain M. (1999). Cancer Patient T Cells Genetically Targeted to Prostate-Specific Membrane Antigen Specifically Lyse Prostate Cancer Cells and Release Cytokines in Response to Prostate-Specific Membrane Antigen. Neoplasia.

[38] Eshhar Z., Waks T., Gross G., Schindler D.G. (1993). Specific activation and targeting of cytotoxic lymphocytes through chimeric single chains consisting of antibody-binding domains and the gamma or zeta subunits of the immunoglobulin and T-cell receptors. Proc. Natl. Acad. Sci. USA.

[39] Goverman J., Gomez S.M., Segesman K.D., Hunkapiller T., Laug W.E., Hood L. (1990). Chimeric immunoglobulin-T cell receptor proteins form functional receptors: Implications for T cell receptor complex formation and activation. Cell.

[40] Ochi T., Maruta M., Tanimoto K., Kondo F., Yamamoto T., Kurata M., Fujiwara H., Masumoto J., Takenaka K., Yasukawa M. (2021). A single-chain antibody generation system yielding CAR-T cells with superior antitumor function. Commun. Biol..

[41] Kang T.H., Seong B.L. (2020). Solubility, Stability, and Avidity of Recombinant Antibody Fragments Expressed in Microorganisms. Front. Microbiol..

[42] Asensio M.A., Lim Y.W., Wayham N., Stadtmiller K., Edgar R.C., Leong J., Leong R., Mizrahi R.A., Adams M.S., Simons J.F. (2019). Antibody repertoire analysis of mouse immunization protocols using microfluidics and molecular genomics. mAbs.

[43] Lonberg N. (2008). Fully human antibodies from transgenic mouse and phage display platforms. Curr. Opin. Immunol..

[44] Zajc C.U., Salzer B., Taft J.M., Reddy S.T., Lehner M., Traxlmayr M.W. (2021). Driving CARs with alternative navigation tools—The potential of engineered binding scaffolds. FEBS J..

[45] Asaadi Y., Jouneghani F.F., Janani S., Rahbarizadeh F. (2021). A comprehensive comparison between camelid nanobodies and single chain variable fragments. Biomark. Res..

[46] Schmidts A., Ormhøj M., Choi B.D., Taylor A.O., Bouffard A.A., Scarfò I., Larson R.C., Frigault M.J., Gallagher K., Castano A.P. (2019). Rational design of a trimeric APRIL-based CAR-binding domain enables efficient targeting of multiple myeloma. Blood Adv..

[47] Wang D., Starr R., Chang W.-C., Aguilar B., Alizadeh D., Wright S.L., Yang X., Brito A., Sarkissian A., Ostberg J.R. (2020). Chlorotoxin-directed CAR T cells for specific and effective targeting of glioblastoma. Sci. Transl. Med..

[48] Jayaraman J., Mellody M.P., Hou A.J., Desai R.P., Fung A.W., Pham A.H.T., Chen Y.Y., Zhao W. (2020). CAR-T design: Elements and their synergistic function. EBioMedicine.

[49] Zhang H., Zhao P., Huang H. (2020). Engineering better chimeric antigen receptor T cells. Exp. Hematol. Oncol..

[50] Ng Y.-Y., Tay J.C., Li Z., Wang J., Zhu J., Wang S. (2020). T Cells Expressing NKG2D CAR with a DAP12 Signaling Domain Stimulate Lower Cytokine Production While Effective in Tumor Eradication. Mol. Ther..

[51] Guedan S., Calderon H., Posey A.D., Maus M.V. (2019). Engineering and Design of Chimeric Antigen Receptors. Mol. Ther. Methods Clin. Dev..

[52] Tyagarajan S., Spencer T., Smith J. (2020). Optimizing CAR-T Cell Manufacturing Processes during Pivotal Clinical Trials. Mol. Ther. Methods Clin. Dev..

[53] Jackson Z., Roe A., Sharma A.A., Lopes F.B.T.P., Talla A., Kleinsorge-Block S., Zamborsky K., Schiavone J., Manjappa S., Schauner R. (2020). Automated Manufacture of Autologous CD19 CAR-T Cells for Treatment of Non-hodgkin Lymphoma. Front. Immunol..

[54] Jürgens B., Clarke N.S. (2019). Evolution of CAR T-cell immunotherapy in terms of patenting activity. Nat. Biotechnol..

[55] Yang J., Kim B., Kim G.Y., Jung G.Y., Seo S.W. (2019). Synthetic biology for evolutionary engineering: From perturbation of genotype to acquisition of desired phenotype. Biotechnol. Biofuels.

[56] Kunjapur A.M., Pfingstag P., Thompson N.C. (2018). Gene synthesis allows biologists to source genes from farther away in the tree of life. Nat. Commun..

[57] Petrenko V.A. (2018). Landscape Phage: Evolution from Phage Display to Nanobiotechnology. Viruses.

[58] Winter G., Harris W.J. (1993). Humanized antibodies. Trends Pharmacol. Sci..

[59] Zinsli L.V., Stierlin N., Loessner M.J., Schmelcher M. (2021). Deimmunization of protein therapeutics—Recent advances in experimental and computational epitope prediction and deletion. Comput. Struct. Biotechnol. J..

[60] Laffleur B., Pascal V., Sirac C., Cogné M. (2012). Production of Human or Humanized Antibodies in Mice. Mol. Radio-Oncol..

[61] Zhou X., Tu S., Wang C., Huang R., Deng L., Song C., Yue C., He Y., Yang J., Liang Z. (2020). Phase I Trial of Fourth-Generation Anti-CD19 Chimeric Antigen Receptor T Cells Against Relapsed or Refractory B Cell Non-Hodgkin Lymphomas. Front. Immunol..

[62] Chmielewski M., Kopecky C., Hombach A.A., Abken H. (2011). IL-12 Release by Engineered T Cells Expressing Chimeric Antigen Receptors Can Effectively Muster an Antigen-Independent Macrophage Response on Tumor Cells That Have Shut Down Tumor Antigen Expression. Cancer Res..

[63] Eyquem J., Mansilla-Soto J., Giavridis T., van der Stegen S.J.C., Hamieh M., Cunanan K.M., Odak A., Gönen M., Sadelain M. (2017). Targeting a CAR to the TRAC locus with CRISPR/Cas9 enhances tumour rejection. Nature.

[64] Kim D.W., Cho J.-Y. (2020). Recent Advances in Allogeneic CAR-T Cells. Biomolecules.

[65] Savoldo B., Ramos C.A., Liu E., Mims M.P., Keating M.J., Carrum G., Kamble R.T., Bollard C.M., Gee A.P., Mei Z. (2011). CD28 costimulation improves expansion and persistence of chimeric antigen receptor–modified T cells in lymphoma patients. J. Clin. Investig..

[66] Maher J., Brentjens R.J., Gunset G., Rivière I., Sadelain M. (2002). Human T-lymphocyte cytotoxicity and proliferation directed by a single chimeric TCRζ/CD28 receptor. Nat. Biotechnol..

[67] Xie G., Dong H., Liang Y., Ham J.D., Rizwan R., Chen J. (2020). CAR-NK cells: A promising cellular immunotherapy for cancer. EBioMedicine.

[68] Davila M.L., Brentjens R.J. (2016). CD19-Targeted CAR T cells as novel cancer immunotherapy for relapsed or refractory B-cell acute lymphoblastic leukemia. Clin. Adv. Hematol. Oncol. HO.

[69] Sommermeyer D., Hudecek M., Kosasih P.L., Gogishvili T., Maloney D.G., Turtle C.J., Riddell S.R. (2016). Chimeric antigen receptor-modified T cells derived from defined CD8+ and CD4+ subsets confer superior antitumor reactivity in vivo. Leukemia.

[70] Xu X., Huang S., Xiao X., Sun Q., Liang X., Chen S., Zhao Z., Huo Z., Tu S., Li Y. (2021). Challenges and Clinical Strategies of CAR T-Cell Therapy for Acute Lymphoblastic Leukemia: Overview and Developments. Front. Immunol..

[71] Hartmann J., Schüßler-Lenz M., Bondanza A., Buchholz C.J. (2017). Clinical development of CAR T cells—challenges and opportunities in translating innovative treatment concepts. EMBO Mol. Med..

[72] Mariuzza R.A., Agnihotri P., and Orban J. (2020). The structural basis of T-cell receptor (TCR) activation: An enduring enigma. J. Biol. Chem..

[73] Li R., Ma C., Cai H., Chen W. (2020). The CAR T-Cell Mechanoimmunology at a Glance. Adv. Sci..

[74] Davenport A.J., Cross R.S., Watson K.A., Liao Y., Shi W., Prince H.M., Beavis P.A., Trapani J.A., Kershaw M.H., Ritchie D.S. (2018). Chimeric antigen receptor T cells form nonclassical and potent immune synapses driving rapid cytotoxicity. Proc. Natl. Acad. Sci. USA.

[75] Papadopoulou M., Tieppo P., McGovern N., Gosselin F., Chan J.K.Y., Goetgeluk G., Dauby N., Cogan A., Donner C., Ginhoux F. (2019). TCR Sequencing Reveals the Distinct Development of Fetal and Adult Human Vγ9Vδ2 T Cells. J. Immunol..

[76] Waldman A.D., Fritz J.M., Lenardo M.J. (2020). A guide to cancer immunotherapy: From T cell basic science to clinical practice. Nat. Rev. Immunol..

[77] Scheuermann R., Racila E. (1995). CD19 Antigen in Leukemia and Lymphoma Diagnosis and Immunotherapy. Leuk. Lymphoma.

[78] Maude S.L., Teachey D., Porter D.L., Grupp S.A. (2015). CD19-targeted chimeric antigen receptor T-cell therapy for acute lymphoblastic leukemia. Blood.

[79] Hammer O. (2012). CD19 as an attractive target for antibody-based therapy. mAbs.

[80] Spiegel J.Y., Patel S., Muffly L., Hossain N.M., Oak J., Baird J.H., Frank M.J., Shiraz P., Sahaf B., Craig J. (2021). CAR T cells with dual targeting of CD19 and CD22 in adult patients with recurrent or refractory B cell malignancies: A phase 1 trial. Nat. Med..

[81] Ginaldi L., De Martinis M., Matutes E., Farahat N., Morilla R., Catovsky D. (1998). Levels of expression of CD19 and CD20 in chronic B cell leukaemias. J. Clin. Pathol..

[82] Seidel U.J.E., Schlegel P., Grosse-Hovest L., Hofmann M., Aulwurm S., Pyz E., Schuster F.R., Meisel R., Ebinger M., Feuchtinger T. (2016). Reduction of Minimal Residual Disease in Pediatric B-lineage Acute Lymphoblastic Leukemia by an Fc-optimized CD19 Antibody. Mol. Ther..

[83] Qin H., Ramakrishna S., Nguyen S., Fountaine T.J., Ponduri A., Stetler-Stevenson M., Yuan C.M., Haso W., Shern J.F., Shah N.N. (2018). Preclinical Development of Bivalent Chimeric Antigen Receptors Targeting Both CD19 and CD22. Mol. Ther. Oncolytics.

[84] Fry T.J., Shah N.N., Orentas R.J., Stetler-Stevenson M., Yuan C.M., Ramakrishna S., Wolters P., Martin S., Delbrook C., Yates B. (2018). CD22-targeted CAR T cells induce remission in B-ALL that is naive or resistant to CD19-targeted CAR immunotherapy. Nat. Med..

[85] Watanabe K., Terakura S., Martens A.C., van Meerten T., Uchiyama S., Imai M., Sakemura R., Goto T., Hanajiri R., Imahashi N. (2015). Target Antigen Density Governs the Efficacy of Anti–CD20-CD28-CD3 ζ Chimeric Antigen Receptor–Modified Effector CD8^+^ T Cells. J. Immunol..

[86] Majzner R.G., Rietberg S.P., Sotillo E., Dong R., Vachharajani V.T., Labanieh L., Myklebust J.H., Kadapakkam M., Weber E.W., Tousley A.M. (2020). Tuning the Antigen Density Requirement for CAR T-cell Activity. Cancer Discov..

[87] Ormhøj M., Scarfò I., Cabral M.L., Bailey S., Lorrey S.J., Bouffard A.A., Castano A.P., Larson R.C., Riley L.S., Schmidts A. (2019). Chimeric Antigen Receptor T Cells Targeting CD79b Show Efficacy in Lymphoma with or without Cotargeting CD19. Clin. Cancer Res..

[88] Ding S., Mao X., Cao Y., Wang N., Xu H., Zhou J. (2020). Targeting CD79b for Chimeric Antigen Receptor T-Cell Therapy of B-Cell Lymphomas. Target. Oncol..

[89] Teachey D.T., Hunger S.P. (2021). Anti-CD7 CAR T cells for T-ALL: Impressive early-stage efficacy. Nat. Rev. Clin. Oncol..

[90] Maciocia P., A Wawrzyniecka P., Philip B., Ricciardelli I., Akarca A.U., Onuoha S.C., Legut M., Cole D., Sewell A.K., Gritti G. (2017). Targeting the T cell receptor β-chain constant region for immunotherapy of T cell malignancies. Nat. Med..

[91] Pan J., Tan Y., Wang G., Deng B., Ling Z., Song W., Seery S., Zhang Y., Peng S., Xu J. (2021). Donor-Derived CD7 Chimeric Antigen Receptor T Cells for T-Cell Acute Lymphoblastic Leukemia: First-in-Human, Phase I Trial. J. Clin. Oncol..

[92] Cummins K., Gill S. (2019). Will CAR T cell therapy have a role in AML? Promises and pitfalls. Semin. Hematol..

[93] Gill S., Tasian S., Ruella M., Shestova O., Li Y., Porter D.L., Carroll M., Danet-Desnoyers G., Scholler J., Grupp S.A. (2014). Preclinical targeting of human acute myeloid leukemia and myeloablation using chimeric antigen receptor–modified T cells. Blood.

[94] Kim M.Y., Yu K.-R., Kenderian S.S., Ruella M., Chen S., Shin T.-H., Aljanahi A.A., Schreeder D., Klichinsky M., Shestova O. (2018). Genetic Inactivation of CD33 in Hematopoietic Stem Cells to Enable CAR T Cell Immunotherapy for Acute Myeloid Leukemia. Cell.

[95] Hou A.J., Chen L.C., Chen Y.Y. (2021). Navigating CAR-T cells through the solid-tumour microenvironment. Nat. Rev. Drug Discov..

[96] Teplyakov A., Obmolova G., Luo J., Gilliland G.L. (2018). Crystal structure of B-cell co-receptor CD19 in complex with antibody B43 reveals an unexpected fold. Proteins Struct. Funct. Bioinform..

[97] Ghorashian S., Kramer A.M., Onuoha S., Wright G., Bartram J., Richardson R., Albon S.J., Casanovas-Company J., Castro F., Popova B. (2019). Enhanced CAR T cell expansion and prolonged persistence in pediatric patients with ALL treated with a low-affinity CD19 CAR. Nat. Med..

[98] Long A.H., Haso W.M., Shern J.F., Wanhainen K.M., Murgai M., Ingaramo M., Smith J.P., Walker A.J., Kohler M.E., Venkateshwara V.R. (2015). 4-1BB costimulation ameliorates T cell exhaustion induced by tonic signaling of chimeric antigen receptors. Nat. Med..

[99] Garcia K.C., Degano M., Stanfield R.L., Brunmark A., Jackson M.R., Peterson P.A., Teyton L., Wilson I.A. (1996). An αβ T Cell Receptor Structure at 2.5 υ and Its Orientation in the TCR-MHC Complex. Science.

[100] Garboczi D.N., Ghosh P., Utz U., Fan Q.R., Biddison W.E., Wiley D.C. (1996). Structure of the complex between human T-cell receptor, viral peptide and HLA-A2. Nature.

[101] Watanabe N., Bajgain P., Sukumaran S., Ansari S., Heslop H.E., Rooney C.M., Brenner M.K., Leen A.M., Vera J.F. (2016). Fine-tuning the CAR spacer improves T-cell potency. OncoImmunology.

[102] Long A.H., Haso W.M., Orentas R.J. (2013). Lessons learned from a highly-active CD22-specific chimeric antigen receptor. OncoImmunology.

[103] James S.E., Greenberg P.D., Jensen M.C., Lin Y., Wang J., Till B.G., Raubitschek A.A., Forman S.J., Press O.W. (2008). Antigen Sensitivity of CD22-Specific Chimeric TCR Is Modulated by Target Epitope Distance from the Cell Membrane. J. Immunol..

[104] Haso W., Lee D.W., Shah N.N., Stetler-Stevenson M., Yuan C.M., Pastan I.H., Dimitrov D.S., Morgan R.A., Fitzgerald D.J., Barrett D.M. (2013). Anti-CD22–chimeric antigen receptors targeting B-cell precursor acute lymphoblastic leukemia. Blood.

[105] Möricke A., Ratei R., Ludwig W.-D., Harbott J., Borkhardt A., Viehmann S., Zimmermann M., Gadner H., Riehm H., Schrappe M. (2004). Prognostic Factors in CD10 Negative Precursor B-Cell Acute Lymphoblastic Leukemia in Children: Data from Three Consecutive Trials ALL-BFM 86, 90, and 95. Blood.

[106] Sędek Ł., Bulsa J., Sonsala A., Twardoch M., Wieczorek M., Malinowska I., Derwich K., Niedźwiecki M., Sobol-Milejska G., Kowalczyk J.R. (2014). The immunophenotypes of blast cells in B-cell precursor acute lymphoblastic leukemia: How different are they from their normal counterparts?. Cytom. Part B Clin. Cytom..

[107] Baird J.H., Frank M.J., Craig J., Patel S., Spiegel J.Y., Sahaf B., Oak J.S., Younes S.F., Ozawa M.G., Yang E. (2021). CD22-directed CAR T-cell therapy induces complete remissions in CD19-directed CAR–refractory large B-cell lymphoma. Blood.

[108] Schneider D., Xiong Y., Wu D., Hu P., Alabanza L., Steimle B., Dropulić B. (2021). Trispecific CD19-CD20-CD22-targeting duoCAR-T cells eliminate antigen-heterogeneous B cell tumors in preclinical models. Sci. Transl. Med..

[109] Fousek K., Watanabe J., Joseph S.K., George A., An X., Byrd T.T., Morris J., Luong A., Martínez-Paniagua M.A., Sanber K. (2021). CAR T-cells that target acute B-lineage leukemia irrespective of CD19 expression. Leukemia.

[110] Ramakrishna S., Highfill S.L., Walsh Z., Nguyen S.M., Lei H., Shern J.F., Qin H., Kraft I.L., Stetler-Stevenson M., Yuan C.M. (2019). Modulation of Target Antigen Density Improves CAR T-cell Functionality and Persistence. Clin. Cancer Res..

[111] Tong C., Zhang Y., Liu Y., Ji X., Zhang W., Guo Y., Han X., Ti D., Dai H., Wang C. (2020). Optimized tandem CD19/CD20 CAR-engineered T cells in refractory/relapsed B-cell lymphoma. Blood.

[112] Guo Y., Feng K., Tong C., Jia H., Liu Y., Wang Y., Ti D., Yang Q., Wu Z., Han W. (2020). Efficiency and side effects of anti-CD38 CAR T cells in an adult patient with relapsed B-ALL after failure of bi-specific CD19/CD22 CAR T cell treatment. Cell. Mol. Immunol..

[113] Jyoti N., Maria T., de Regina J.-K., Ruud W.J.R., Pino J.P., Huipin Y., de Bruijn J.D., Ossenkoppele G.J., Zweegman S., Smit L. (2019). CD38 as a therapeutic target for adult acute myeloid leukemia and T-cell acute lymphoblastic leukemia. Haematologica.

[114] Haubner S., Perna F., Köhnke T., Schmidt C., Berman S., Augsberger C., Schnorfeil F.M., Krupka C., Lichtenegger F.S., Liu X. (2019). Coexpression profile of leukemic stem cell markers for combinatorial targeted therapy in AML. Leukemia.

[115] Tambaro F.P., Singh H., Jones E., Rytting M., Mahadeo K.M., Thompson P., Daver N., DiNardo C., Kadia T., Garcia-Manero G. (2021). Autologous CD33-CAR-T cells for treatment of relapsed/refractory acute myelogenous leukemia. Leukemia.

[116] Mardiana S., Gill S. (2020). CAR T Cells for Acute Myeloid Leukemia: State of the Art and Future Directions. Front. Oncol..

[117] Baroni M.L., Martinez D.S., Aguera F.G., Ho H.R., Castella M., Zanetti S., Hernandez T.V., De La Guardia R.D., Castaño J., Anguita E. (2020). 41BB-based and CD28-based CD123-redirected T-cells ablate human normal hematopoiesis in vivo. J. Immunother. Cancer.

[118] Styczyński J., Ebmt F.T.I.D.W.P., Tridello G., Koster L., Iacobelli S., Van Biezen A., Van Der Werf S., Mikulska M., Gil L., Cordonnier C. (2020). Death after hematopoietic stem cell transplantation: Changes over calendar year time, infections and associated factors. Bone Marrow Transplant..

[119] Liu X., Jiang S., Fang C., Yang S., Olalere D., Pequignot E.C., Cogdill A., Li N., Ramones M., Granda B. (2015). Affinity-Tuned ErbB2 or EGFR Chimeric Antigen Receptor T Cells Exhibit an Increased Therapeutic Index against Tumors in Mice. Cancer Res..

[120] Du H., Hirabayashi K., Ahn S., Kren N.P., Montgomery S.A., Wang X., Tiruthani K., Mirlekar B., Michaud D., Greene K. (2019). Antitumor Responses in the Absence of Toxicity in Solid Tumors by Targeting B7-H3 via Chimeric Antigen Receptor T Cells. Cancer Cell.

[121] Xu X., Sun Q., Liang X., Chen Z., Zhang X., Zhou X., Li M., Tu H., Liu Y., Tu S. (2019). Mechanisms of Relapse After CD19 CAR T-Cell Therapy for Acute Lymphoblastic Leukemia and Its Prevention and Treatment Strategies. Front. Immunol..

[122] Kagoya Y., Tanaka S., Guo T., Anczurowski M., Wang C.-H., Saso K., Butler M.O., Minden M.D., Hirano N. (2018). A novel chimeric antigen receptor containing a JAK–STAT signaling domain mediates superior antitumor effects. Nat. Med..

[123] Salter A.I., Rajan A., Kennedy J.J., Ivey R.G., Shelby S.A., Leung I., Templeton M.L., Muhunthan V., Voillet V., Sommermeyer D. (2021). Comparative analysis of TCR and CAR signaling informs CAR designs with superior antigen sensitivity and in vivo function. Sci. Signal..

[124] Watanabe K., Kuramitsu S., Posey A.D., June C.H. (2018). Expanding the Therapeutic Window for CAR T Cell Therapy in Solid Tumors: The Knowns and Unknowns of CAR T Cell Biology. Front. Immunol..

[125] Dong R., A Libby K., Blaeschke F., Fuchs W., Marson A., Vale R.D., Su X. (2020). Rewired signaling network in T cells expressing the chimeric antigen receptor ( CAR ). EMBO J..

[126] Stopfer L.E., Gajadhar A.S., Patel B., Gallien S., Frederick D.T., Boland G.M., Sullivan R.J., White F.M. (2021). Absolute quantification of tumor antigens using embedded MHC-I isotopologue calibrants. Proc. Natl. Acad. Sci. USA.

[127] Croft N., Smith S.A., Wong Y.C., Tan C.T., Dudek N.L., Flesch I.E.A., Lin L.C.W., Tscharke D.C., Purcell A.W. (2013). Kinetics of Antigen Expression and Epitope Presentation during Virus Infection. PLOS Pathog..

[128] Walker A.J., Majzner R.G., Zhang L., Wanhainen K., Long A.H., Nguyen S.M., Lopomo P., Vigny M., Fry T.J., Orentas R.J. (2017). Tumor Antigen and Receptor Densities Regulate Efficacy of a Chimeric Antigen Receptor Targeting Anaplastic Lymphoma Kinase. Mol. Ther..

[129] Heitzeneder S., Bosse K.R., Zhu Z., Zhelev D., Majzner R.G., Radosevich M.T., Dhingra S., Sotillo E., Buongervino S., Pascual-Pasto G. (2022). GPC2-CAR T cells tuned for low antigen density mediate potent activity against neuroblastoma without toxicity. Cancer Cell.

[130] A Morgan R., Yang J.C., Kitano M., E Dudley M., Laurencot C.M., A Rosenberg S. (2010). Case Report of a Serious Adverse Event Following the Administration of T Cells Transduced With a Chimeric Antigen Receptor Recognizing ERBB2. Mol. Ther..

[131] Hill J.A., Giralt S., Torgerson T.R., Lazarus H.M. (2019). CAR-T—And a side order of IgG, to go?—Immunoglobulin replacement in patients receiving CAR-T cell therapy. Blood Rev..

[132] Seitz C.M., Mittelstaet J., Atar D., Hau J., Reiter S., Illi C., Kieble V., Engert F., Drees B., Bender G. (2021). Novel adapter CAR-T cell technology for precisely controllable multiplex cancer targeting. OncoImmunology.

[133] Sommermeyer D., Hill T., Shamah S.M., Salter A., Chen Y., Mohler K.M., Riddell S.R. (2017). Fully human CD19-specific chimeric antigen receptors for T-cell therapy. Leukemia.

[134] Muzard J., Bouabdelli M., Zahid M., Ollivier V., Lacapère J.J., Jandrot-Perrus M., Billiald P. (2009). Design and humanization of a murine scFv that blocks human platelet glycoprotein VI in vitro. FEBS J..

[135] Lam N., Trinklein N.D., Buelow B., Patterson G.H., Ojha N., Kochenderfer J.N. (2020). Anti-BCMA chimeric antigen receptors with fully human heavy-chain-only antigen recognition domains. Nat. Commun..

[136] Holliger P., Hudson P.J. (2005). Engineered antibody fragments and the rise of single domains. Nat. Biotechnol..

[137] Jensen M.C., Popplewell L., Cooper L.J., DiGiusto D., Kalos M., Ostberg J.R., Forman S.J. (2010). Antitransgene Rejection Responses Contribute to Attenuated Persistence of Adoptively Transferred CD20/CD19-Specific Chimeric Antigen Receptor Redirected T Cells in Humans. Biol. Blood Marrow Transplant..

[138] Wang Y., Li C., Xia J., Li P., Cao J., Pan B., Tan X., Li H., Qi K., Wang X. (2021). Humoral immune reconstitution after anti-BCMA CAR T-cell therapy in relapsed/refractory multiple myeloma. Blood Adv..

[139] Doan A., Pulsipher M.A. (2018). Hypogammaglobulinemia due to CAR T-cell therapy. Pediatr. Blood Cancer.

[140] Chen X., Jensen P.E. (2008). The role of B lymphocytes as antigen-presenting cells. Arch. Immunol. Ther. Exp..

[141] Harris D.T., Kranz D.M. (2016). Adoptive T Cell Therapies: A Comparison of T Cell Receptors and Chimeric Antigen Receptors. Trends Pharmacol. Sci..

[142] De Groot A.S., Terry F., Cousens L., Martin W. (2013). Beyond humanization and de-immunization: Tolerization as a method for reducing the immunogenicity of biologics. Expert Rev. Clin. Pharmacol..

[143] Augustyniak D., Majkowska-Skrobek G., Roszkowiak J., Dorotkiewicz-Jach A. (2017). Defensive and Offensive Cross-Reactive Antibodies Elicited by Pathogens: The Good, the Bad and the Ugly. Curr. Med. Chem..

[144] Polymeros D., Bogdanos D.P., Day R., Arioli D., Vergani D., Forbes A. (2006). Does Cross-Reactivity between Mycobacterium avium paratuberculosis and Human Intestinal Antigens Characterize Crohn’s Disease?. Gastroenterology.

[145] Larmonier C.B., Shehab K.W., Ghishan F.K., Kiela P. (2015). T Lymphocyte Dynamics in Inflammatory Bowel Diseases: Role of the Microbiome. BioMed Res. Int..

[146] Bartelds G.M., Krieckaert C.L.M., Nurmohamed M.T., van Schouwenburg P.A., Lems W.F., Twisk J.W.R., Dijkmans B.A.C., Aarden L., jan Wolbink G. (2011). Development of Antidrug Antibodies Against Adalimumab and Association With Disease Activity and Treatment Failure During Long-term Follow-up. JAMA.

[147] Abramson J.S., Palomba M.L., Gordon L.I., Lunning M.A., Wang M., Arnason J., Mehta A., Purev E., Maloney D.G., Andreadis C. (2020). Lisocabtagene maraleucel for patients with relapsed or refractory large B-cell lymphomas (TRANSCEND NHL 001): A multicentre seamless design study. Lancet.

[148] Wang M., Munoz J., Goy A., Locke F.L., Jacobson C.A., Hill B.T., Timmerman J.M., Holmes H., Jaglowski S., Flinn I.W. (2020). KTE-X19 CAR T-Cell Therapy in Relapsed or Refractory Mantle-Cell Lymphoma. N. Engl. J. Med..

[149] Maude S.L., Laetsch T.W., Buechner J., Rives S., Boyer M., Bittencourt H., Bader P., Verneris M.R., Stefanski H.E., Myers G.D. (2018). Tisagenlecleucel in Children and Young Adults with B-Cell Lymphoblastic Leukemia. N. Engl. J. Med..

[150] Munshi N.C., Anderson L.D., Shah N., Madduri D., Berdeja J., Lonial S., Raje N., Lin Y., Siegel D., Oriol A. (2021). Idecabtagene Vicleucel in Relapsed and Refractory Multiple Myeloma. N. Engl. J. Med..

[151] Park J.H., Geyer M.B., Brentjens R.J. (2016). CD19-targeted CAR T-cell therapeutics for hematologic malignancies: Interpreting clinical outcomes to date. Blood.

[152] Kenderian S., Ruella M., Shestova O., Klichinsky M., Aikawa V., Morrissette J.J.D., Scholler J., Song D., Porter D.L., Carroll M.C. (2015). CD33-specific chimeric antigen receptor T cells exhibit potent preclinical activity against human acute myeloid leukemia. Leukemia.

[153] Zhong Q., Zhu Y.-M., Zheng L.-L., Shen H.-J., Ou R.-M., Liu Z., She Y.-L., Chen R., Li C., Huang J. (2018). Chimeric Antigen Receptor-T Cells with 4-1BB Co-Stimulatory Domain Present a Superior Treatment Outcome than Those with CD28 Domain Based on Bioinformatics. Acta Haematol..

[154] Shao Z., Schwarz H. (2011). CD137 ligand, a member of the tumor necrosis factor family, regulates immune responses via reverse signal transduction. J. Leukoc. Biol..

[155] Esensten J.H., Helou Y.A., Chopra G., Weiss A., Bluestone J.A. (2016). CD28 Costimulation: From Mechanism to Therapy. Immunity.

[156] Colombetti S., Basso V., Mueller D., Mondino A. (2006). Prolonged TCR/CD28 Engagement Drives IL-2-Independent T Cell Clonal Expansion through Signaling Mediated by the Mammalian Target of Rapamycin. J. Immunol..

[157] Zhao Z., Condomines M., Van Der Stegen S.J.C., Perna F., Kloss C.C., Gunset G., Plotkin J., Sadelain M. (2015). Structural Design of Engineered Costimulation Determines Tumor Rejection Kinetics and Persistence of CAR T Cells. Cancer Cell.

[158] Kawalekar O.U., O’Connor R.S., Fraietta J.A., Guo L., Mcgettigan S.E., Posey A.D., Patel P.R., Guedan S., Scholler J., Keith B. (2016). Distinct Signaling of Coreceptors Regulates Specific Metabolism Pathways and Impacts Memory Development in CAR T Cells. Immunity.

[159] Ying Z., He T., Wang X., Zheng W., Lin N., Tu M., Xie Y., Ping L., Zhang C., Liu W. (2019). Parallel Comparison of 4-1BB or CD28 Co-stimulated CD19-Targeted CAR-T Cells for B Cell Non-Hodgkin’s Lymphoma. Mol. Ther. Oncolytics.

[160] Drent E., Poels R., Ruiter R., Van De Donk N.W.C.J., Zweegman S., Yuan H., de Bruijn J., Sadelain M., Lokhorst H.M., Groen R.W.J. (2019). Combined CD28 and 4-1BB Costimulation Potentiates Affinity-tuned Chimeric Antigen Receptor–engineered T Cells. Clin. Cancer Res..

[161] Li G., Boucher J.C., Kotani H., Park K., Zhang Y., Shrestha B., Wang X., Guan L., Beatty N., Abate-Daga D. (2018). 4-1BB enhancement of CAR T function requires NF-κB and TRAFs. JCI Insight.

[162] Porter D.L., Hwang W.-T., Frey N.V., Lacey S.F., Shaw P.A., Loren A.W., Bagg A., Marcucci K.T., Shen A., Gonzalez V. (2015). Chimeric antigen receptor T cells persist and induce sustained remissions in relapsed refractory chronic lymphocytic leukemia. Sci. Transl. Med..

[163] Park J.H., Rivière I., Gonen M., Wang X., Sénéchal B., Curran K.J., Sauter C., Wang Y., Santomasso B., Mead E. (2018). Long-Term Follow-up of CD19 CAR Therapy in Acute Lymphoblastic Leukemia. N. Engl. J. Med..

[164] Gökbuget N., Stanze D., Beck J., Diedrich H., Horst H.-A., Hüttmann A., Kobbe G., Kreuzer K.-A., Leimer L., Reichle A. (2012). Outcome of relapsed adult lymphoblastic leukemia depends on response to salvage chemotherapy, prognostic factors, and performance of stem cell transplantation. Blood.

[165] Martin A., Morgan E., Hijiya N. (2012). Relapsed or refractory pediatric acute lymphoblastic leukemia: Current and emerging treatments. Paediatr. Drugs.

[166] Sun W., Malvar J., Sposto R., Verma A., Wilkes J.J., Dennis R., Heym K., Laetsch T.W., Widener M., Rheingold S.R. (2018). Outcome of children with multiply relapsed B-cell acute lymphoblastic leukemia: A therapeutic advances in childhood leukemia & lymphoma study. Leukemia.

[167] Locatelli F., Whitlock J.A., Peters C., Chen-Santel C., Chia V., Dennis R.M., Heym K.M., Katz A.J., Kelsh M.A., Sposto R. (2020). Blinatumomab versus historical standard therapy in pediatric patients with relapsed/refractory Ph-negative B-cell precursor acute lymphoblastic leukemia. Leukemia.

[168] Parker K.R., Migliorini D., Perkey E., Yost K.E., Bhaduri A., Bagga P., Haris M., Wilson N.E., Liu F., Gabunia K. (2020). Single-Cell Analyses Identify Brain Mural Cells Expressing CD19 as Potential Off-Tumor Targets for CAR-T Immunotherapies. Cell.

[169] Mejstrikova E., Klinger M., Markovic A., Zugmaier G., Locatelli F. (2021). CD19 expression in pediatric patients with relapsed/refractory B-cell precursor acute lymphoblastic leukemia pre- and post-treatment with blinatumomab. Pediatr. Blood Cancer.

[170] Neelapu S.S. (2019). CAR-T efficacy: Is conditioning the key?. Blood.

[171] Harrison R.P., Rafiq Q.A., Medcalf N. (2018). Centralised versus decentralised manufacturing and the delivery of healthcare products: A United Kingdom exemplar. Cytotherapy.

[172] Maude S.L. (2017). Future directions in chimeric antigen receptor T cell therapy. Curr. Opin. Pediatr..

[173] Gardner R.A., Finney O., Annesley C., Brakke H., Summers C., Leger K., Bleakley M., Brown C., Mgebroff S., Kelly-Spratt K.S. (2017). Intent-to-treat leukemia remission by CD19 CAR T cells of defined formulation and dose in children and young adults. Blood.

[174] Pasquini M.C., Hu Z.-H., Curran K., Laetsch T., Locke F., Rouce R., Pulsipher M.A., Phillips C.L., Keating A., Frigault M.J. (2020). Real-world evidence of tisagenlecleucel for pediatric acute lymphoblastic leukemia and non-Hodgkin lymphoma. Blood Adv..

[175] (2021). EHA2021 Virtual Congress Abstract Book. HemaSphere.

[176] Diorio C., Maude S.L. (2020). CAR T cells vs allogeneic HSCT for poor-risk ALL. Hematology.

[177] Gaynon P.S., Harris R.E., Altman A.J., Bostrom B.C., Breneman J.C., Hawks R., Steele D., Zipf T., Stram D.O., Villaluna D. (2006). Bone Marrow Transplantation Versus Prolonged Intensive Chemotherapy for Children With Acute Lymphoblastic Leukemia and an Initial Bone Marrow Relapse Within 12 Months of the Completion of Primary Therapy: Children’s Oncology Group Study CCG-1941. J. Clin. Oncol..

[178] Locatelli F., Zugmaier G., Mergen N., Bader P., Jeha S., Schlegel P.-G., Bourquin J.-P., Handgretinger R., Brethon B., Rössig C. (2022). Blinatumomab In pediatric relapsed/refractory B-cell acute lymphoblastic leukemia: RIALTO expanded access study final analysis. Blood Adv..

[179] Jiang H., Hu Y., Mei H. (2020). Consolidative allogeneic hematopoietic stem cell transplantation after chimeric antigen receptor T-cell therapy for relapsed/refractory B-cell acute lymphoblastic leukemia: Who? When? Why?. Biomark. Res..

[180] Hierlmeier S., Eyrich M., Wölfl M., Schlegel P.-G., Wiegering V. (2018). Early and late complications following hematopoietic stem cell transplantation in pediatric patients—A retrospective analysis over 11 years. PLoS ONE.

[181] Shah N.N., Highfill S.L., Shalabi H., Yates B., Jin J., Wolters P.L., Ombrello A., Steinberg S.M., Martin S., Delbrook C. (2020). CD4/CD8 T-Cell Selection Affects Chimeric Antigen Receptor (CAR) T-Cell Potency and Toxicity: Updated Results From a Phase I Anti-CD22 CAR T-Cell Trial. J. Clin. Oncol..

[182] Lee D.W., A Gardner R., Porter D.L., Louis C.U., Ahmed N., Jensen M.C., Grupp S.A., Mackall C.L. (2014). Current concepts in the diagnosis and management of cytokine release syndrome. Blood.

[183] Hay K.A., Hanafi L.-A., Li D., Gust J., Liles W.C., Wurfel M.M., López J.A., Chen J., Chung D., Harju-Baker S. (2017). Kinetics and biomarkers of severe cytokine release syndrome after CD19 chimeric antigen receptor–modified T-cell therapy. Blood.

[184] Fitzgerald J.C., Weiss S., Maude S.L., Barrett D.M., Lacey S.F., Melenhorst J.J., Shaw P., Berg R.A., June C.H., Porter D.L. (2017). Cytokine Release Syndrome After Chimeric Antigen Receptor T Cell Therapy for Acute Lymphoblastic Leukemia. Crit. Care Med..

[185] Neelapu S.S., Tummala S., Kebriaei P., Wierda W., Gutierrez C., Locke F.L., Komanduri K.V., Lin Y., Jain N., Daver N. (2018). Chimeric antigen receptor T-cell therapy—Assessment and management of toxicities. Nat. Rev. Clin. Oncol..

[186] Wei J., Liu Y., Wang C., Zhang Y., Tong C., Dai G., Wang W., Rasko J., Melenhorst J.J., Qian W. (2020). The model of cytokine release syndrome in CAR T-cell treatment for B-cell non-Hodgkin lymphoma. Signal Transduct. Target. Ther..

[187] Gust J., Hay K.A., Hanafi L.-A., Li D., Myerson D., Gonzalez-Cuyar L.F., Yeung C., Liles W.C., Wurfel M., Lopez J.A. (2017). Endothelial Activation and Blood–Brain Barrier Disruption in Neurotoxicity after Adoptive Immunotherapy with CD19 CAR-T Cells. Cancer Discov..

[188] Siegler E.L., Kenderian S.S. (2020). Neurotoxicity and Cytokine Release Syndrome after Chimeric Antigen Receptor T Cell Therapy: Insights Into Mechanisms and Novel Therapies. Front. Immunol..

[189] Gust J., Ponce R., Liles W.C., Garden G.A., Turtle C.J. (2020). Cytokines in CAR T Cell–Associated Neurotoxicity. Front. Immunol..

[190] Nellan A., McCully C.M.L., Garcia R.C., Jayaprakash N., Widemann B.C., Lee D.W., Warren K.E. (2018). Improved CNS exposure to tocilizumab after cerebrospinal fluid compared to intravenous administration in rhesus macaques. Blood.

[191] Riegler L.L., Jones G.P., Lee D.W. (2019). Current approaches in the grading and management of cytokine release syndrome after chimeric antigen receptor T-cell therapy. Ther. Clin. Risk Manag..

[192] Wudhikarn K., Bansal R., Khurana A., Hathcock M., Ruff M., Carabenciov I.D., Braksick S.A., Bennani N.N., Paludo J., Villasboas J.C. (2021). Characteristics, outcomes, and risk factors of ICANS after axicabtagene ciloleucel: Does age matter?. J. Clin. Oncol..

[193] Sievers S., Watson G., Johncy S., Adkins S. (2020). Recognizing and Grading CAR T-Cell Toxicities: An Advanced Practitioner Perspective. Front. Oncol..

[194] Zhao X., Yang J., Zhang X., Lu X.-A., Xiong M., Zhang J., Zhou X., Qi F., He T., Ding Y. (2020). Efficacy and Safety of CD28- or 4-1BB-Based CD19 CAR-T Cells in B Cell Acute Lymphoblastic Leukemia. Mol. Ther. Oncolytics.

[195] (2018). JCAR015 in ALL: A Root-Cause Investigation. Cancer Discov..

[196] Henderson L.A., Cron R.Q. (2020). Macrophage Activation Syndrome and Secondary Hemophagocytic Lymphohistiocytosis in Childhood Inflammatory Disorders: Diagnosis and Management. Pediatr. Drugs.

[197] Voskoboinik I., Whisstock J.C., Trapani J.A. (2015). Perforin and granzymes: Function, dysfunction and human pathology. Nat. Rev. Immunol..

[198] Gadoury-Levesque V., Dong L., Su R., Chen J., Zhang K., Risma K.A., Marsh R.A., Sun M. (2020). Frequency and spectrum of disease-causing variants in 1892 patients with suspected genetic HLH disorders. Blood Adv..

[199] Grom A.A., Horne A., De Benedetti F. (2016). Macrophage activation syndrome in the era of biologic therapy. Nat. Rev. Rheumatol..

[200] Canna S.W., de Jesus A.A., Gouni S., Brooks S.R., Marrero B., Liu Y., DiMattia M.A., Zaal K.J.M., Sanchez G.A.M., Kim H. (2014). An activating NLRC4 inflammasome mutation causes autoinflammation with recurrent macrophage activation syndrome. Nat. Genet..

[201] Teachey D.T., Rheingold S.R., Maude S.L., Zugmaier G., Barrett D.M., Seif A.E., Nichols K.E., Suppa E.K., Kalos M., Berg R.A. (2013). Cytokine release syndrome after blinatumomab treatment related to abnormal macrophage activation and ameliorated with cytokine-directed therapy. Blood.

[202] Morris E.C., Neelapu S.S., Giavridis T., Sadelain M. (2021). Cytokine release syndrome and associated neurotoxicity in cancer immunotherapy. Nat. Rev. Immunol..

[203] A Misbah S., Weeratunga P. (2020). Immunoglobulin replacement and quality of life after CAR T-cell therapy. Lancet Oncol..

[204] Hill J.A., Seo S.K. (2020). How I prevent infections in patients receiving CD19-targeted chimeric antigen receptor T cells for B-cell malignancies. Blood.

[205] Rabilloud T., Potier D., Pankaew S., Nozais M., Loosveld M., Payet-Bornet D. (2021). Single-cell profiling identifies pre-existing CD19-negative subclones in a B-ALL patient with CD19-negative relapse after CAR-T therapy. Nat. Commun..

[206] Weiland J., Pal D., Case M., Irving J., Ponthan F., Koschmieder S., Heidenreich O., Von Stackelberg A., Eckert C., Vormoor J. (2016). BCP-ALL blasts are not dependent on CD19 expression for leukaemic maintenance. Leukemia.

[207] Dourthe M.-E., Rabian F., Yakouben K., Chevillon F., Cabannes-Hamy A., Méchinaud F., Grain A., Chaillou D., Rahal I., Caillat-Zucman S. (2021). Determinants of CD19-positive vs CD19-negative relapse after tisagenlecleucel for B-cell acute lymphoblastic leukemia. Leukemia.

[208] Sotillo E., Barrett D.M., Black K.L., Bagashev A., Oldridge D., Wu G., Sussman R., LaNauze C., Ruella M., Gazzara M.R. (2015). Convergence of Acquired Mutations and Alternative Splicing of CD19 Enables Resistance to CART-19 Immunotherapy. Cancer Discov..

[209] Gardner R., Wu D., Cherian S., Fang M., Hanafi L.-A., Finney O., Smithers H., Jensen M.C., Riddell S.R., Maloney D.G. (2016). Acquisition of a CD19-negative myeloid phenotype allows immune escape of MLL-rearranged B-ALL from CD19 CAR-T-cell therapy. Blood.

[210] Zhou Y., You M.J., Young K.H., Lin P., Lu G., Medeiros L.J., Bueso-Ramos C.E. (2012). Advances in the molecular pathobiology of B-lymphoblastic leukemia. Hum. Pathol..

[211] Salvaris R., Fedele P. (2021). Targeted Therapy in Acute Lymphoblastic Leukaemia. J. Pers. Med..

[212] Zhang W.-Y., Liu Y., Wang Y., Wang C.-M., Yang Q.-M., Zhu H.-L., Han W.-D. (2017). Long-term safety and efficacy of CART-20 cells in patients with refractory or relapsed B-cell non-Hodgkin lymphoma: 5-years follow-up results of the phase I and IIa trials. Signal Transduct. Target. Ther..

[213] Liang A., Ye S., Li P., Huang J., Zhu S., Yao X., Zhou L., Xu Y., Zhu J., Zheng C. (2021). Safety and efficacy of a novel anti-CD20 chimeric antigen receptor (CAR)-T cell therapy in relapsed/refractory (r/r) B-cell non-Hodgkin lymphoma (B-NHL) patients after failing CD19 CAR-T therapy. J. Clin. Oncol..

[214] Shadman M., Yeung C., Redman M., Lee S.Y., Lee D.H., Ra S., Ujjani C.S., Dezube B.J., Poh C., Warren E.H. (2021). Safety and Efficacy of Third Generation CD20 Targeted CAR-T (MB-106) for Treatment of Relapsed/Refractory B-NHL and CLL. Blood.

[215] Schultz L.M., Muffly L.S., Spiegel J.Y., Ramakrishna S., Hossain N., Baggott C., Sahaf B., Patel S., Craig J., Yoon J. (2019). Phase I Trial Using CD19/CD22 Bispecific CAR T Cells in Pediatric and Adult Acute Lymphoblastic Leukemia (ALL). Blood.

[216] Shalabi H., Yates B., Shahani S., Qin H., HIghfill S.L., Panch S., Tran M., Stroncek D., Hoffman L., Little L. (2020). Abstract CT051: Safety and efficacy of CD19/CD22 CAR T cells in children and young adults with relapsed/refractory ALL. Cancer Res..

[217] Shah N.N., Johnson B.D., Schneider D., Zhu F., Szabo A., Keever-Taylor C.A., Krueger W., Worden A.A., Kadan M.J., Yim S. (2020). Bispecific anti-CD20, anti-CD19 CAR T cells for relapsed B cell malignancies: A phase 1 dose escalation and expansion trial. Nat. Med..

[218] Köksal H., Dillard P., Josefsson S.E., Maggadottir S.M., Pollmann S., Fåne A., Blaker Y.N., Beiske K., Huse K., Kolstad A. (2019). Preclinical development of CD37CAR T-cell therapy for treatment of B-cell lymphoma. Blood Adv..

[219] Scarfò I., Ormhøj M., Frigault M.J., Castano A.P., Lorrey S., Bouffard A.A., Van Scoyk A., Rodig S.J., Shay A.J., Aster J.C. (2018). Anti-CD37 chimeric antigen receptor T cells are active against B- and T-cell lymphomas. Blood.

[220] Patterson J.D., Henson J.C., Breese R.O., Bielamowicz K.J., Rodriguez A. (2020). CAR T Cell Therapy for Pediatric Brain Tumors. Front. Oncol..

[221] Richards R.M., Sotillo E., Majzner R.G. (2018). CAR T Cell Therapy for Neuroblastoma. Front. Immunol..

[222] Majzner R.G., Theruvath J.L., Nellan A., Heitzeneder S., Cui Y., Mount C.W., Rietberg S.P., Linde M.H., Xu P., Rota C. (2019). CAR T Cells Targeting B7-H3, a Pan-Cancer Antigen, Demonstrate Potent Preclinical Activity Against Pediatric Solid Tumors and Brain Tumors. Clin. Cancer Res..

[223] Raje N., Berdeja J., Lin Y., Siegel D., Jagannath S., Madduri D., Liedtke M., Rosenblatt J., Maus M.V., Turka A. (2019). Anti-BCMA CAR T-Cell Therapy bb2121 in Relapsed or Refractory Multiple Myeloma. N. Engl. J. Med..

[224] Qin H., Yang L., Chukinas J.A., Shah N.N., Tarun S., Pouzolles M., Chien C.D., Niswander L.M., Welch A.R., Taylor N.A. (2021). Systematic preclinical evaluation of CD33-directed chimeric antigen receptor T cell immunotherapy for acute myeloid leukemia defines optimized construct design. J. Immunother. Cancer.

[225] Wolf P., Alzubi J., Gratzke C., Cathomen T. (2021). The potential of CAR T cell therapy for prostate cancer. Nat. Rev. Urol..

[226] Akce M., Zaidi M.Y., Waller E.K., El-Rayes B.F., Lesinski G.B. (2018). The Potential of CAR T Cell Therapy in Pancreatic Cancer. Front. Immunol..

[227] Bo M.D., De Mattia E., Baboci L., Mezzalira S., Cecchin E., Assaraf Y.G., Toffoli G. (2020). New insights into the pharmacological, immunological, and CAR-T-cell approaches in the treatment of hepatocellular carcinoma. Drug Resist. Updat..

[228] Kudo K., Imai C., Lorenzini P., Kamiya T., Kono K., Davidoff A.M., Chng W.J., Campana D. (2014). T Lymphocytes Expressing a CD16 Signaling Receptor Exert Antibody-Dependent Cancer Cell Killing. Cancer Res..

[229] Rataj F., Jacobi S.J., Stoiber S., Asang F., Ogonek J., Tokarew N., Cadilha B., Van Puijenbroek E., Heise C., Duewell P. (2019). High-affinity CD16-polymorphism and Fc-engineered antibodies enable activity of CD16-chimeric antigen receptor-modified T cells for cancer therapy. Br. J. Cancer.

[230] Tamada K., Geng D., Sakoda Y., Bansal N., Srivastava R., Li Z., Davila E. (2012). Redirecting Gene-Modified T Cells toward Various Cancer Types Using Tagged Antibodies. Clin. Cancer Res..

[231] Ma J.S.Y., Kim J.Y., Kazane S.A., Choi S.-H., Yun H.Y., Kim M.S., Rodgers D.T., Pugh H.M., Singer O., Sun S.B. (2016). Versatile strategy for controlling the specificity and activity of engineered T cells. Proc. Natl. Acad. Sci. USA.

[232] Rodgers D.T., Mazagova M., Hampton E.N., Cao Y., Ramadoss N.S., Hardy I.R., Schulman A., Du J., Wang F., Singer O. (2016). Switch-mediated activation and retargeting of CAR-T cells for B-cell malignancies. Proc. Natl. Acad. Sci. USA.

[233] Bachmann M. (2019). The UniCAR system: A modular CAR T cell approach to improve the safety of CAR T cells. Immunol. Lett..

[234] Mu J., Edwards J., Zaritskaya L., Swers J., Gupta A., LaFleur D., Hilbert D., Richman L. (2020). Selective targeting of HER2-overexpressing solid tumors with a next-generation CAR-T cell therapy. J. Clin. Oncol..

[235] Grote S., Mittelstaet J., Baden C., Chan K.C., Seitz C., Schlegel P., Kaiser A., Handgretinger R., Schleicher S. (2020). Adapter chimeric antigen receptor (AdCAR)-engineered NK-92 cells: An off-the-shelf cellular therapeutic for universal tumor targeting. Oncoimmunology.

[236] Urbanska K., Lanitis E., Poussin M., Lynn R.C., Gavin B.P., Kelderman S., Yu J., Scholler N., Powell D.J. (2012). A Universal Strategy for Adoptive Immunotherapy of Cancer through Use of a Novel T-cell Antigen Receptor. Cancer Res..

[237] Cho J.H., Collins J.J., Wong W.W. (2018). Universal Chimeric Antigen Receptors for Multiplexed and Logical Control of T Cell Responses. Cell.

[238] Kim M.S., Ma J.S.Y., Yun H., Cao Y., Kim J.Y., Chi V., Wang D., Woods A., Sherwood L., Caballero D. (2015). Redirection of Genetically Engineered CAR-T Cells Using Bifunctional Small Molecules. J. Am. Chem. Soc..

[239] Ryman J.T., Meibohm B. (2017). Pharmacokinetics of Monoclonal Antibodies. CPT Pharmacomet. Syst. Pharmacol..

[240] Waldmann T.A., Strober W. (1969). Metabolism of Immunoglobulins. Prog. Allergy.

[241] Knauf M.J., Bell D.P., Hirtzer P., Luo Z.P., Young J.D., Katre N.V. (1988). Relationship of effective molecular size to systemic clearance in rats of recombinant interleukin-2 chemically modified with water-soluble polymers. J. Biol. Chem..

[242] Lampson L.A. (2011). Monoclonal antibodies in neuro-oncology: Getting past the blood-brain barrier. mAbs.

[243] Cavaco M., Gaspar D., Castanho M.A., Neves V. (2020). Antibodies for the Treatment of Brain Metastases, a Dream or a Reality?. Pharmaceutics.

[244] Cruz E., Kayser V. (2019). Monoclonal antibody therapy of solid tumors: Clinical limitations and novel strategies to enhance treatment efficacy. Biol. Targets Ther..

[245] Davis K.L., Mackall C.L. (2016). Immunotherapy for acute lymphoblastic leukemia: From famine to feast. Blood Adv..

[246] Lenk L., Alsadeq A., Schewe D.M. (2020). Involvement of the central nervous system in acute lymphoblastic leukemia: Opinions on molecular mechanisms and clinical implications based on recent data. Cancer Metastasis Rev..

[247] Abid H., Watthanasuntorn K., Shah O., Gnanajothy R. (2019). Efficacy of Pembrolizumab and Nivolumab in Crossing the Blood Brain Barrier. Cureus.

[248] Cartellieri M., Feldmann A., Koristka S., Arndt C., Loff S., Ehninger A.V., Von Bonin M., Bejestani E.P., Ehninger G., Bachmann M.P. (2016). Switching CAR T cells on and off: A novel modular platform for retargeting of T cells to AML blasts. Blood Cancer J..

[249] Goebeler M.-E., Knop S., Viardot A., Kufer P., Topp M.S., Einsele H., Noppeney R., Hess G., Kallert S., Mackensen A. (2016). Bispecific T-Cell Engager (BiTE) Antibody Construct Blinatumomab for the Treatment of Patients With Relapsed/Refractory Non-Hodgkin Lymphoma: Final Results From a Phase I Study. J. Clin. Oncol..

[250] . Hong W.X., Haebe S., Lee A.S., Westphalen C.B., Norton J.A., Jiang W., Levy R. (2020). Intratumoral Immunotherapy for Early-stage Solid Tumors. Clin. Cancer Res..

[251] Adusumilli P.S., Zauderer M.G., Rivière I., Solomon S.B., Rusch V.W., O’Cearbhaill R.E., Zhu A., Cheema W., Chintala N.K., Halton E. (2021). A Phase I Trial of Regional Mesothelin-Targeted CAR T-cell Therapy in Patients with Malignant Pleural Disease, in Combination with the Anti–PD-1 Agent Pembrolizumab. Cancer Discov..

[252] Choi B.D., O’Rourke D.M., Maus M.V. (2017). Engineering Chimeric Antigen Receptor T cells to Treat Glioblastoma. Int. J. Target. Ther. Cancer.

[253] Schulz H., Pels H., Schmidt-Wolf I., Zeelen U., Germing U., Engert A. (2004). Intraventricular treatment of relapsed central nervous system lymphoma with the anti-CD20 antibody rituximab. Haematology.

[254] Sison E.A.R., Silverman L.B. (2014). CNS prophylaxis in pediatric acute lymphoblastic leukemia. Hematology.

[255] Ravandi F., Walter R.B., Subklewe M., Buecklein V., Jongen-Lavrencic M., Paschka P., Ossenkoppele G.J., Kantarjian H.M., Hindoyan A., Agarwal S.K. (2020). Updated results from phase I dose-escalation study of AMG 330, a bispecific T-cell engager molecule, in patients with relapsed/refractory acute myeloid leukemia (R/R AML). J. Clin. Oncol..

[256] Krupka C., Kufer P., Kischel R., Zugmaier G., Bögeholz J., Köhnke T., Lichtenegger F.S., Schneider S., Metzeler K., Fiegl M. (2014). CD33 target validation and sustained depletion of AML blasts in long-term cultures by the bispecific T-cell–engaging antibody AMG 330. Blood.

[257] Wang X., Chang W.-C., Wong C.W., Colcher D., Sherman M., Ostberg J.R., Forman S.J., Riddell S.R., Jensen M.C. (2011). A transgene-encoded cell surface polypeptide for selection, in vivo tracking, and ablation of engineered cells. Blood.

[258] Di Stasi A., Tey S.-K., Dotti G., Fujita Y., Kennedy-Nasser A., Martinez C., Straathof K., Liu E., Durett A.G., Grilley B. (2011). Inducible Apoptosis as a Safety Switch for Adoptive Cell Therapy. N. Engl. J. Med..

[259] Budde L.E., Berger C., Lin Y., Wang J., Lin X., Frayo S.E., Brouns S.A., Spencer D.M., Till B.G., Jensen M.C. (2013). Combining a CD20 Chimeric Antigen Receptor and an Inducible Caspase 9 Suicide Switch to Improve the Efficacy and Safety of T Cell Adoptive Immunotherapy for Lymphoma. PLoS ONE.

[260] Sterner R.M., Sakemura R., Cox M.J., Yang N., Khadka R.H., Forsman C.L., Hansen M.J., Jin F., Ayasoufi K., Hefazi M. (2019). GM-CSF inhibition reduces cytokine release syndrome and neuroinflammation but enhances CAR-T cell function in xenografts. Blood.

[261] Roybal K.T., Rupp L.J., Morsut L., Walker W.J., McNally K.A., Park J.S., Lim W.A. (2016). Precision Tumor Recognition by T Cells With Combinatorial Antigen-Sensing Circuits. Cell.

[262] Kloss C.C., Condomines M., Cartellieri M., Bachmann M., Sadelain M. (2013). Combinatorial antigen recognition with balanced signaling promotes selective tumor eradication by engineered T cells. Nat. Biotechnol..

[263] Zajc C.U., Dobersberger M., Schaffner I., Mlynek G., Pühringer D., Salzer B., Djinović-Carugo K., Steinberger P., Linhares A.D.S., Yang N.J. (2020). A conformation-specific ON-switch for controlling CAR T cells with an orally available drug. Proc. Natl. Acad. Sci. USA.

[264] Park S., Pascua E., Lindquist K.C., Kimberlin C., Deng X., Mak Y.S.L., Melton Z., Johnson T.O., Lin R., Boldajipour B. (2021). Direct control of CAR T cells through small molecule-regulated antibodies. Nat. Commun..

[265] Weber E.W., Lynn R.C., Sotillo E., Lattin J., Xu P., Mackall C.L. (2019). Pharmacologic control of CAR-T cell function using dasatinib. Blood Adv..

[266] Mestermann K., Giavridis T., Weber J., Rydzek J., Frenz S., Nerreter T., Mades A., Sadelain M., Einsele H., Hudecek M. (2019). The tyrosine kinase inhibitor dasatinib acts as a pharmacologic on/off switch for CAR T cells. Sci. Transl. Med..

[267] Deng Q., Han G., Puebla-Osorio N., Ma M.C.J., Strati P., Chasen B., Dai E., Dang M., Jain N., Yang H. (2020). Characteristics of anti-CD19 CAR T cell infusion products associated with efficacy and toxicity in patients with large B cell lymphomas. Nat. Med..

[268] Li-Weber M., Krammer P.H. (2003). Regulation of IL4 gene expression by T cells and therapeutic perspectives. Nat. Rev. Immunol..

[269] Mohammed S., Sukumaran S., Bajgain P., Watanabe N., Heslop H.E., Rooney C.M., Brenner M.K., Fisher W.E., Leen A.M., Vera J.F. (2017). Improving Chimeric Antigen Receptor-Modified T Cell Function by Reversing the Immunosuppressive Tumor Microenvironment of Pancreatic Cancer. Mol. Ther..

[270] Kloss C.C., Lee J., Zhang A., Chen F., Melenhorst J.J., Lacey S.F., Maus M.V., Fraietta J.A., Zhao Y., June C.H. (2018). Dominant-Negative TGF-beta Receptor Enhances PSMA-Targeted Human CAR T Cell Proliferation And Augments Prostate Cancer Eradication. Mol. Ther..

[271] Liu X., Ranganathan R., Jiang S., Fang C., Sun J., Kim S., Newick K., Lo A., June C.H., Zhao Y. (2016). A Chimeric Switch-Receptor Targeting PD1 Augments the Efficacy of Second-Generation CAR T Cells in Advanced Solid Tumors. Cancer Res..

[272] Rafiq S., Yeku O.O., Jackson H.J., Purdon T.J., Van Leeuwen D.G., Drakes D.J., Song M., Miele M.M., Li Z., Wang P. (2018). Targeted delivery of a PD-1-blocking scFv by CAR-T cells enhances anti-tumor efficacy in vivo. Nat. Biotechnol..

[273] Suarez E.R., de Chang K., Sun J., Sui J., Freeman G.J., Signoretti S., Zhu Q., Marasco W.A. (2016). Chimeric antigen receptor T cells secreting anti-PD-L1 antibodies more effectively regress renal cell carcinoma in a humanized mouse model. Oncotarget.

[274] Cherkassky L., Morello A., Villena-Vargas J., Feng Y., Dimitrov D.S., Jones D.R., Sadelain M., Adusumilli P.S. (2016). Human CAR T cells with cell-intrinsic PD-1 checkpoint blockade resist tumor-mediated inhibition. J. Clin. Investig..

[275] Rupp L.J., Schumann K., Roybal K.T., Gate R.E., Ye C.J., Lim W.A., Marson A. (2017). CRISPR/Cas9-mediated PD-1 disruption enhances anti-tumor efficacy of human chimeric antigen receptor T cells. Sci. Rep..

[276] Wang Z., Li N., Feng K., Chen M., Zhang Y., Liu Y., Yang Q., Nie J., Tang N., Zhang X. (2021). Phase I study of CAR-T cells with PD-1 and TCR disruption in mesothelin-positive solid tumors. Cell. Mol. Immunol..

[277] Choi B.D., Yu X., Castano A.P., Darr H., Henderson D.B., Bouffard A.A., Larson R.C., Scarfò I., Bailey S.R., Gerhard G.M. (2019). CRISPR-Cas9 disruption of PD-1 enhances activity of universal EGFRvIII CAR T cells in a preclinical model of human glioblastoma. J. Immunother. Cancer.

[278] Poirot L., Philip B., Schiffer-Mannioui C., Le Clerre D., Chion-Sotinel I., Derniame S., Potrel P., Bas C., Lemaire L., Galetto R. (2015). Multiplex Genome-Edited T-cell Manufacturing Platform for “Off-the-Shelf” Adoptive T-cell Immunotherapies. Cancer Res..

[279] Riley J.L. (2009). PD-1 signaling in primary T cells. Immunol. Rev..

[280] Stenger D., Stief T.A., Kaeuferle T., Willier S., Rataj F., Schober K., Vick B., Lotfi R., Wagner B., Grünewald T.G.P. (2020). Endogenous TCR promotes in vivo persistence of CD19-CAR-T cells compared to a CRISPR/Cas9-mediated TCR knockout CAR. Blood.

[281] Roth T.L., Puig-Saus C., Yu R., Shifrut E., Carnevale J., Li P.J., Hiatt J., Saco J., Krystofinski P., Li H. (2018). Reprogramming human T cell function and specificity with non-viral genome targeting. Nature.

[282] MacLeod D.T., Antony J., Martin A.J., Moser R.J., Hekele A., Wetzel K.J., Brown A.E., Triggiano M.A., Hux J.A., Pham C.D. (2017). Integration of a CD19 CAR into the TCR Alpha Chain Locus Streamlines Production of Allogeneic Gene-Edited CAR T Cells. Mol. Ther..

[283] Liu Y., Liu G., Wang J., Zheng Z.-Y., Jia L., Rui W., Huang D., Zhou Z.-X., Zhou L., Wu X. (2021). Chimeric STAR receptors using TCR machinery mediate robust responses against solid tumors. Sci. Transl. Med..

[284] Baeuerle P.A., Ding J., Patel E., Thorausch N., Horton H., Gierut J., Scarfo I., Choudhary R., Kiner O., Krishnamurthy J. (2019). Synthetic TRuC receptors engaging the complete T cell receptor for potent anti-tumor response. Nat. Commun..

[285] Koneru M., Purdon T.J., Spriggs D., Koneru S., Brentjens R.J. (2015). IL-12 secreting tumor-targeted chimeric antigen receptor T cells eradicate ovarian tumors in vivo. OncoImmunology.

[286] Hu B., Ren J., Luo Y., Keith B., Young R.M., Scholler J., Zhao Y., June C.H. (2017). Augmentation of Antitumor Immunity by Human and Mouse CAR T Cells Secreting IL-18. Cell Rep..

[287] Hurton L.V., Singh H., Najjar A.M., Switzer K.C., Mi T., Maiti S., Olivares S., Rabinovich B., Huls H., Forget M.-A. (2016). Tethered IL-15 augments antitumor activity and promotes a stem-cell memory subset in tumor-specific T cells. Proc. Natl. Acad. Sci. USA.

[288] Majzner R.G., Mackall C.L. (2019). Clinical lessons learned from the first leg of the CAR T cell journey. Nat. Med..

[289] Sterner R.C., Sterner R.M. (2021). CAR-T cell therapy: Current limitations and potential strategies. Blood Cancer J..

[290] Yang J., He J., Zhang X., Wang Z., Zhang Y., Cai S., Sun Z., Ye X., He Y., Shen L. (2019). A Feasibility and Safety Study of a New CD19-Directed Fast CAR-T Therapy for Refractory and Relapsed B Cell Acute Lymphoblastic Leukemia. Blood.

[291] Whittington M.D., McQueen R.B., Ollendorf D.A., Kumar V.M., Chapman R.H., Tice J., Pearson S.D., Campbell J.D. (2018). Long-term Survival and Value of Chimeric Antigen Receptor T-Cell Therapy for Pediatric Patients With Relapsed or Refractory Leukemia. JAMA Pediatr..

[292] Sarkar R.R., Gloude N.J., Schiff D., Murphy J.D. (2019). Cost-Effectiveness of Chimeric Antigen Receptor T-Cell Therapy in Pediatric Relapsed/Refractory B-Cell Acute Lymphoblastic Leukemia. JNCI J. Natl. Cancer Inst..

